# Single-cell spatiotemporal dissection of the human maternal–fetal interface

**DOI:** 10.1038/s41586-026-10316-x

**Published:** 2026-04-08

**Authors:** Cheng Wang, Yan Zhou, Yuejun Wang, Tuhin Kumar Guha, Zhida Luo, Anxhela Mustafaraj, Tara I. McIntyre, Marisa E. Schwab, Brittany R. Davidson, Gabriella C. Reeder, Ronald J. Wong, Sarah K. England, Juan M. Gonzalez, Robert Blelloch, Alexis J. Combes, Linda C. Giudice, Adrian Erlebacher, Tippi C. MacKenzie, David K. Stevenson, Gary M. Shaw, Michael P. Snyder, Xiaofei Sun, Virginia D. Winn, Susan J. Fisher, Jingjing Li

**Affiliations:** 1 Eli and Edythe Broad Center of Regeneration Medicine and Stem Cell Research, UCSF School of Medicine, University of California San Francisco, San Francisco, CA USA; 2 Department of Neurology, UCSF School of Medicine, University of California San Francisco, San Francisco, CA USA; 3 Bakar Computational Health Sciences Institute, University of California San Francisco, San Francisco, CA USA; 4 Center for Reproductive Sciences, Department of Obstetrics, Gynecology and Reproductive Sciences, University of California San Francisco, San Francisco, CA USA; 5 The Institute for Human Genetics, University of California San Francisco, San Francisco, CA USA; 6 Department of Obstetrics, Gynecology and Reproductive Sciences, UCSF School of Medicine, University of California San Francisco, San Francisco, CA USA; 7 Department of Genetics, Stanford University School of Medicine, Stanford, CA USA; 8 Reproductive Sciences Center, Division of Developmental Biology, Cincinnati Children’s Hospital, Cincinnati, OH USA; 9 Department of Surgery, UCSF School of Medicine, University of California San Francisco, San Francisco, CA USA; 10 UCSF CoLabs, University of California San Francisco, San Francisco, CA USA; 11 Department of Pathology, University of California San Francisco, San Francisco, CA USA; 12 Department of Pediatrics, Stanford University School of Medicine, Stanford, CA USA; 13 Center for Reproductive Health Sciences, Department of Obstetrics and Gynecology, Washington University School of Medicine, St. Louis, MO USA; 14 Department of Urology, UCSF School of Medicine, University of California San Francisco, San Francisco, CA USA; 15 Bakar ImmunoX Initiative, University of California San Francisco, San Francisco, CA USA; 16 Department of Laboratory Medicine, University of California San Francisco, San Francisco, CA USA; 17 The Center for Maternal–Fetal Precision Medicine, UCSF School of Medicine, University of California San Francisco, San Francisco, CA USA; 18 College of Medicine, University of Cincinnati, Cincinnati, OH USA; 19 Department of Obstetrics and Gynecology, Stanford University School of Medicine, Stanford, CA USA

**Keywords:** Genomics, Development, Developmental biology, Computational biology and bioinformatics, Reproductive biology

## Abstract

The human maternal–fetal interface is characterized by mosaic intermingling of maternal and fetal cells^1^. Yet the underlying cellular, molecular and spatial programmes remain incompletely defined. Here we generate a comprehensive atlas of the human maternal–fetal interface across normal pregnancies from early gestation to term by integrating large-scale paired single-nucleus transcriptomic and chromatin accessibility profiling with submicrometre-resolution spatial transcriptomics and CODEX multiplex protein imaging^2^, substantially boosting the spatiotemporal resolution of prior research^3^. This framework delineates common and transient cell types, states and spatial niches across the fetal and maternal compartments, reconstructs transcriptional programmes that guide cytotrophoblast and decidual stromal cell differentiation, and resolves recurrent architecture structural units that build this interface. We identify previously unrecognized arterial endothelial state transitions during cytotrophoblast-mediated spiral artery remodelling and develop a machine learning model that predicts cytotrophoblast invasiveness from transcriptomic signatures. We further discover a decidual stromal cell subtype that suppresses cytotrophoblast invasion via endocannabinoid signalling at the human maternal–fetal interface. By integrating the atlas with genome-wide association data, we pinpoint maternal and fetal cells that are most vulnerable to pre-eclampsia, preterm birth or miscarriage. This resource provides a comprehensive spatially resolved single-cell multiomic reference of the human placenta and decidua and offers a framework for decoding their normal and disordered development.

## Abstract (web summary)

A single-cell multiomic atlas of the human maternal–fetal interface across pregnancy reveals cell types, states and spatial niches, developmental tissue architectures and transcriptional programmes, and identifies cell types with roles in pre-eclampsia, spontaneous preterm birth and miscarriage.

## Main

The human maternal–fetal interface (MFI) is a transient hemi-allogeneic amalgam in which maternal decidual stromal cells (DSCs) support placental attachment, recruit immune cells and create a tolerogenic milieu for patterning fetal cytotrophoblast invasion^1^. During placental development, fetal villous cytotrophoblasts (VCTs) in floating villi fuse into syncytiotrophoblasts (SCTs)^1^, which mediate nutrient and waste exchange and hormone and growth factor secretion, and limit fetal cortisol exposure^1,4^. Alternatively, they form anchoring villi with cell columns that generate invasive extravillous trophoblasts (EVTs)^1^. EVTs invade the decidua and uterine spiral arteries. By the end of the first trimester, EVT remodelling of these vessels establishes low-resistance arteries that enable high-velocity blood flow to the placenta^1^. Previous single-cell studies have been confined to limited gestational windows^3,5–9^ (Supplementary Table 1); here we generate a reference of the human MFI from early gestation to term across normal pregnancies by integrating single-nucleus multiomics with submicrometre spatial mapping. This framework catalogues diverse cell types, resolves transient states and trajectories, maps intercellular communication in situ, and pinpoints vulnerable cell states in pregnancy complications.

## A single-cell map of the interface

**Fig. 1:**
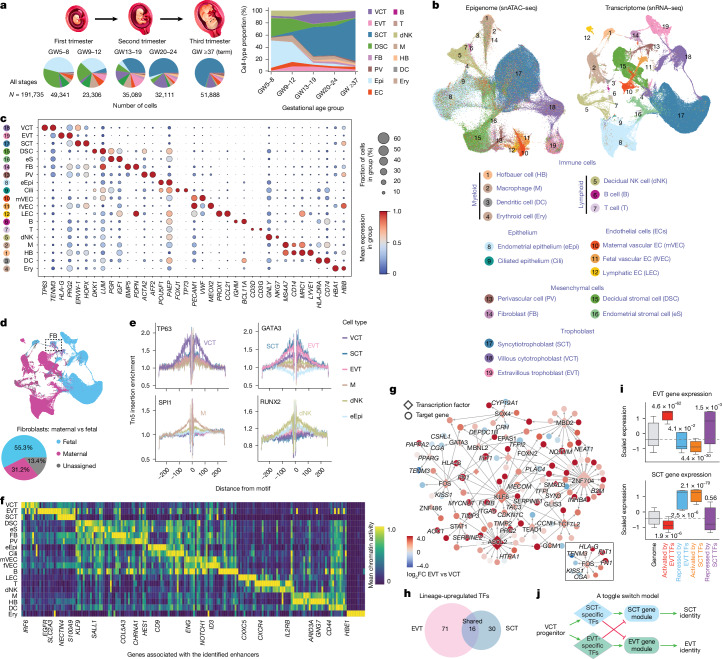
Overview of single-cell multiomics data from the human MFI across gestation. **a**, Experimental design. Nuclei (191,735) from the human MFI were profiled spanning five intervals from early gestation to term. *n* represents the number of nuclei per group. Pie charts show major cell-type proportions in each group. Right, stacked area plot illustrating cell-type composition changes over gestation (cell type abbreviations defined in key below **b**). Image credit: YG Studio/Shutterstock.com. **b**, UMAP projections of snATAC–seq (left) and snRNA-seq (right) for 19 broadly identified cell types (key). **c**, Expression (snRNA-seq) dot plot of selected marker genes across the 19 cell types. Dot size represents the percentage of expressing cells. **d**, Assignment as maternal or fetal for each nucleus by genotype phasing. Bottom, pie chart illustrating the proportion of maternal or fetal fibroblasts. **e**, ATAC–seq footprinting identifies binding sites of cell-type-enriched transcription factors: *TP63* (VCT), *GATA3* (EVT), *SPI1* (M) and *RUNX2* (dNK). Binding sites are marked by depletion of Tn5 insertion flanking the motifs. **f**, Chromatin accessibility heat map showing the activity of the ten most active cell-type-specific enhancers. Regions analysed were cap analysis gene expression (CAGE)-validated enhancers from the FAMTOM5 collection^13^. **g**, Rewiring of GRNs during VCT differentiation into EVT and SCT lineages. The reconstructed EVT GRN is shown. Red and blue denote activation and repression, respectively, relative to VCTs. Inset, a FOS-mediated subnetwork. The top transcription factors with the highest ranked regulatory interactions are shown as examples. **h**, Venn diagram showing uniquely upregulated transcription factors (TFs) in EVTs or SCTs compared to VCTs. **i**, Expression patterns of transcription factors and their target genes reflect the coordinated regulation across two trophoblast lineages. EVT transcription factors activated EVT genes and repressed SCT genes in EVTs, with the reciprocal pattern in SCTs. In box plots, the centre line is the median, box represents the interquartile range (IQR; 25th to 75th percentile), and whiskers show minima and maxima (0.5× IQR) end-points; outliers are not displayed. *P* values, two-tailed Wilcoxon rank-sum tests after Benjamini–Hochberg correction. **j**, Schematic toggle switch model illustrating trophoblast fate reinforcement and suppression of the alternative programme. Source data

**Extended Data Fig. 1:**
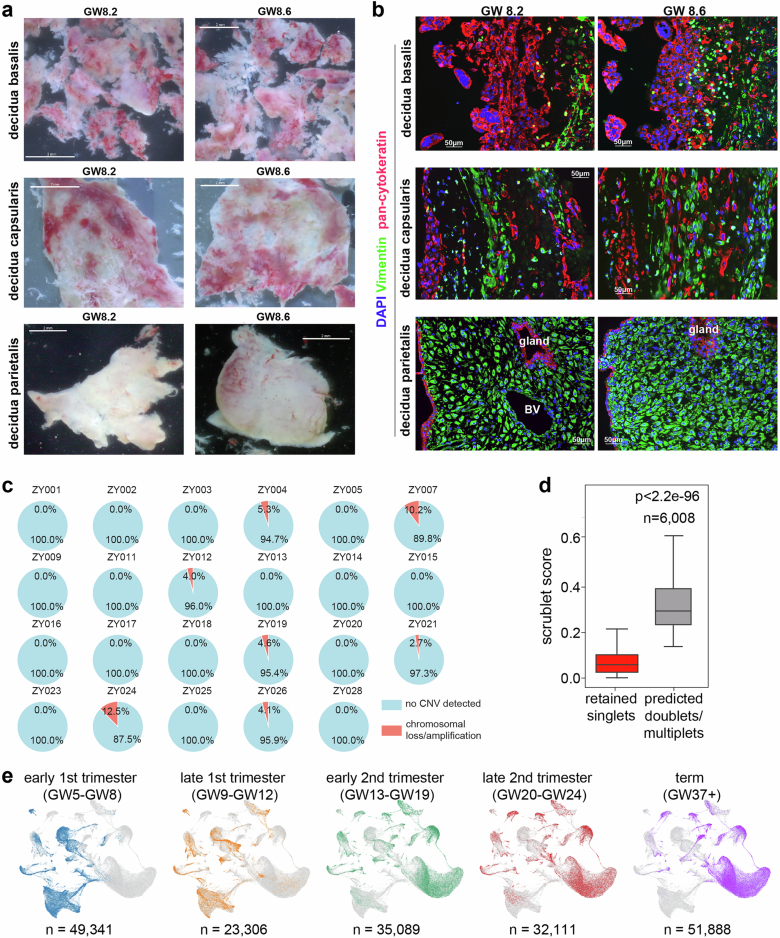
Sample validation and quality-controlled single-nucleus (sn) multi-omic integration. (**a**) Representative brightfield images of early gestation decidua basalis, decidua capsularis and decidua parietalis (left, GW 8.2; right, GW 8.6). Samples from decidua basalis were collected for snMultiome library preparation. (**b**) Immunostaining of Vimentin (green) and pan-cytokeratin (CK, red), highlighted the distinct anatomical structure of decidua basalis vs. other decidual regions (left, GW 8.2; right, GW 8.6). CK^+^ trophoblasts confirmed decidua basalis identity. (**c**) Proportion of fetal cells with putative copy number variations (CNVs) inferred from snATAC-seq using Copy-scAT. No significant germline CNVs (>25% of cells) were detected. (**d**) Scrublet score distributions for predicted singlets and doublets/multiplets; the predicted doublets/multiplets (n = 6,008) were excluded from downstream analyses. Box plots, center line=median, box=IQR (25^th^-75^th^ percentile), minima/maxima=5%/95% of data range; P value, two-tailed Wilcoxon rank-sum test. (**e**) UMAP of nuclei grouped by five gestational stages: early (GW 5–8) and late (GW 9–12) first trimester, early (GW 13–18) and late (GW 19–24) second trimester, and term. Source data

Our single-nucleus multiomics profiling targeted the human MFI from known normal (term gestation) or presumed normal (early/mid gestation) pregnancies (Methods; Supplementary Table 1). Samples encompassed the decidua basalis with embedded EVTs (early gestation) and the basal plate (mid/late gestation) from gestational week (GW) 5 to 39 (Fig. 1a). Sample collection, inspection and validation are detailed in Methods (Extended Data Fig. 1a–d). We performed paired single-nucleus RNA-sequencing (snRNA-seq) and single-nucleus assay for transposase-accessible chromatin with high-throughput sequencing (snATAC–seq) using the 10x Genomics platform, yielding high-quality datasets of 221,380 nuclei (snRNA-seq) and 210,191 nuclei (snATAC–seq) after stringent quality control (Methods). Among these, 191,735 nuclei had paired snRNA-seq and snATAC–seq profiles (Supplementary Table 1). On average, 8,336 nuclei were multiomically profiled per sample. The distribution of sequenced nuclei across gestational week intervals is shown in Fig. 1a and Extended Data Fig. 1e.

**Extended Data Fig. 2:**
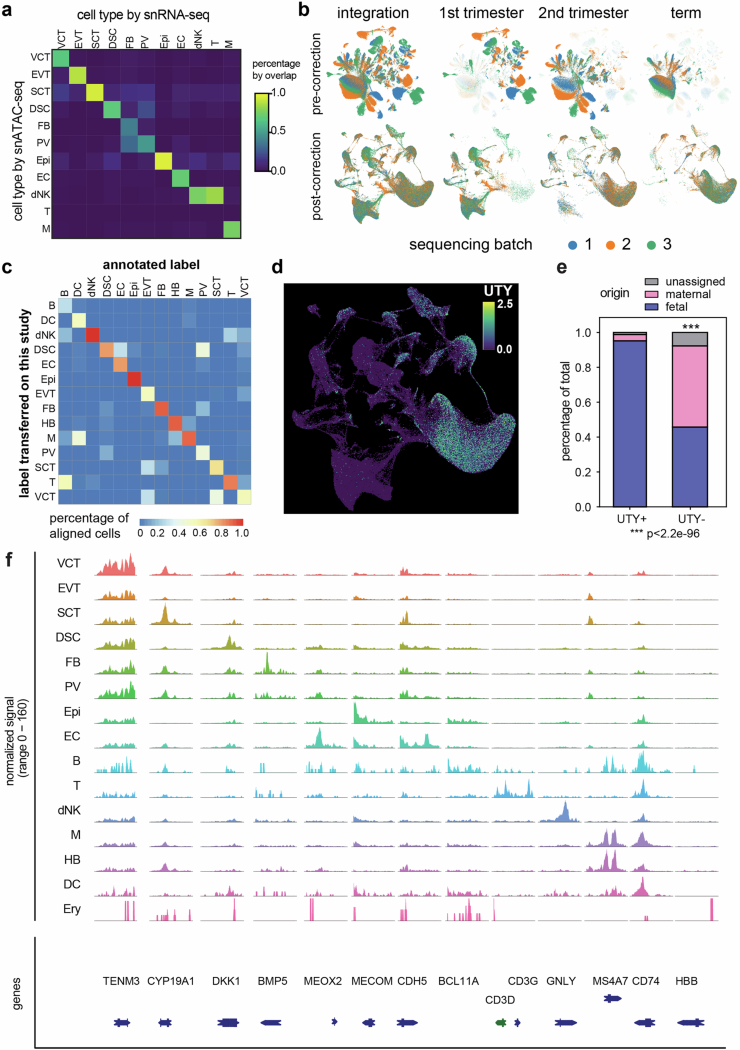
Cell origin assignment and cross-modality annotation. (**a**) Heatmap of concordance between snATAC-seq and snRNA-seq cell type annotations based on gene activity and expression, respectively; colors, fraction of cells with consistent labels. (**b**) UMAPs before and after integration showing effective batch effect correction across three sequencing batches for all gestational age groups. (**c**) Heatmap showing concordance between published annotations in Arutyunyan et al.^3^ and label-transfer predictions by this study. Colors, percentage of cells with matched labels. (**d**) UMAPs showing Y chromosome gene *UTY* expression across 191,735 nuclei. (**e**) Benchmarking Souporcell-based origin assignment by *UTY* expression. Maternal and fetal cell distributions were stratified by *UTY* positivity in pregnancies with male fetuses; 94.3% of *UTY*^+^ cells were classified as fetal (two-tailed χ^2^ test, ***, P < 2.2e-96). (**f**) Cell type-specific ATAC-seq peaks at promoters of canonical marker genes in each cell type, shown as normalized ATAC-seq signal across ±10 kb of transcription start sites. Source data

We annotated cell clusters in the open-chromatin epigenome and transcriptome spaces (Fig. 1b) on the basis of known markers (Supplementary Table 2), which showed strong cell-type specificity (Fig. 1c) and overall concordance between the two modalities (Extended Data Fig. 2a). To confirm annotation accuracy, we verified batch correction (Extended Data Fig. 2b) and performed label transfer by projecting our cell-type annotations onto a prior placental atlas^3^, yielding concordant assignments across major cell classes (Extended Data Fig. 2c). Notably, our study identified many more novel cell subtypes and states. Figure 1a summarizes cell-type composition across developmental windows. Because microanatomy (especially spiral artery distribution) varies across biopsy sites, cross-sample cell-type proportion comparisons can be misleading without spatial context. We therefore emphasized within-cell-type molecular profiles and performed compositional analyses only when spatial information was available or required.

We used Souporcell^10^ to assign maternal versus fetal origin for more than 95% of cells (182,773 out of 191,735; Fig. 1d), validated by Y chromosome markers (Extended Data Fig. 2d,e). Some cell types contained both origins (for example, fibroblasts, FB in Fig. 1d), whereas vascular endothelial cells formed distinct maternal and fetal clusters (Fig. 1b), indicating molecular divergence.

## Cell-type-specific gene regulation

**Extended Data Fig. 3:**
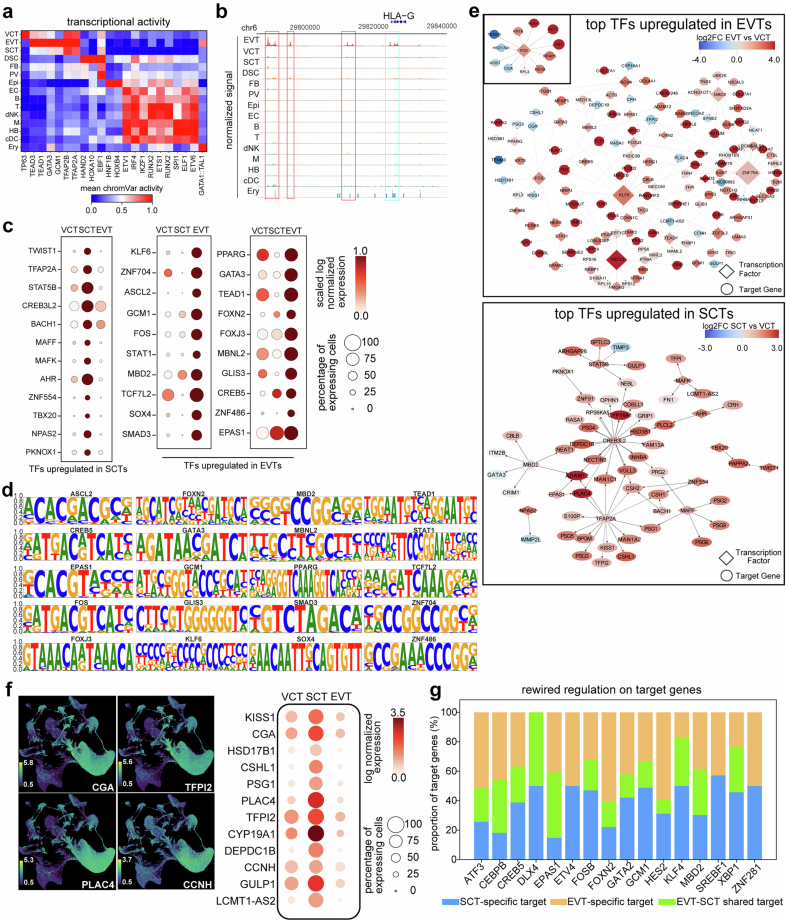
Decoding gene regulation underlying trophoblast differentiation. (**a**) Heatmap of chromVAR activity of cell type-specific TFs. The representative highly expressed and specific TFs/cell-type are shown. (**b**) Chromatin accessibility at the *HLA-G* locus (chr6:29,790,000–29,840,000) across major cell types. Three potential EVT-specific distal enhancer clusters (red) are located within 30 kb upstream of the *HLA-G* promoter (cyan). (**c**) Representative transcription factors (TFs) upregulated in SCTs/EVTs relative to VCTs. (**d**) DNA-binding motifs of the 20 most highly upregulated TFs during VCT-to-EVT transition, shown as PWM logos. (**e**) Gene regulatory networks (GRNs) underlying VCT-to-EVT (upper panel) and VCT-to-SCT (lower panel) differentiation. (**f**) UMAP and dot plots showing that EVT TFs negatively targeted genes in EVTs, many of which had preferential expression in SCTs. (**g**) Bar charts showing the proportion of shared target genes regulated by 16 TFs in EVT and SCT GRNs. The 16 TFs regulate distinct set of target genes despite upregulation during both VCT-to-EVT and VCT-to-SCT transition. Source data

Cell-type-specific genes aligned with cell-type-specific promoter assay for ATAC–seq peaks (Extended Data Fig. 2f). Using chromVar^11^, we inferred transcription factor-binding motifs enriched in ATAC–seq pseudobulk peaks in each cell type (Extended Data Fig. 3a). snATAC–seq footprinting^12^ further resolved transcription factor-binding sites of approximately 20 bp in size within pseudobulk peaks (Fig. 1e). We then mapped open-chromatin peaks to experimentally defined enhancers^13^. The top ten most enriched *cis*-enhancers for each cell type are shown in Fig. 1f (Supplementary Table 2). At the *HLA-G* locus (an EVT marker), we detected an EVT-specific promoter peak and three upstream open-chromatin peaks, including a known enhancer about 10 kb upstream^14^ and two putative distal enhancers (Extended Data Fig. 3b). Together, these analyses revealed extensive cell-type-specific regulatory rewiring.

## A toggle switch model for EVT fate

We used CellOracle^15^ to reconstruct gene regulatory networks (GRNs) in each cell type by integrating snATAC–seq and snRNA-seq data. We focused on the trophoblast lineage to identify regulatory rewiring that directs progenitor VCTs towards terminal fates: EVTs or SCTs. Compared to VCTs, we identified 71 and 30 transcription factors that were specifically upregulated in EVTs and SCTs, respectively (false discovery rate (FDR) ≤ 0.01; top transcription factors in Fig. 1g and Extended Data Fig. 3c; representative motifs in Extended Data Fig. 3d; EVT–SCT overlap in Fig. 1h; full lists in Supplementary Table 3; Methods). CellOracle inferred target genes for these transcription factors. In EVTs, the 20 most highly upregulated transcription factors included *ASCL2*, *FOS*, *KLF6* and *STAT1*, known regulators of VCT-to-EVT differentiation^16–19^, which mediated 167 high-confidence regulatory interactions (strength coefficients >0.1) with 116 target genes (Fig. 1g, Extended Data Fig. 3e and Supplementary Table 3). Among these, FOS positively regulated canonical EVT genes such as *HLA-G*, *KRT8* and *FN1* (Fig. 1g, inset). The same transcription factors activated in EVTs also showed negative associations with genes, which, as expected, were down-regulated in EVTs, but many, in fact, were known SCT markers (for example, *CGA*, *TFPI2* and *PLAC4*; Fig. 1g and Extended Data Fig. 3f). Genome-wide, genes that were positively associated with EVT transcription factors were upregulated in EVTs, whereas negatively associated targets were suppressed in EVTs but enriched in SCTs (Fig. 1i). In EVTs, these negative associations between EVT transcription factors and SCT genes, predicted as putative repressive interactions, are consistent with recent reports that EVT-specific transcription factors functionally suppress the SCT programme in EVTs^16,20,21^. The same analysis of SCT-specific transcription factors showed the reciprocal pattern: activating the SCT programme while negatively associating with EVT-enriched genes in SCTs (Fig. 1i). Together, these observations suggest a bistable toggle switch model that enforces commitment to either the EVT or SCT lineage, locking cells into one fate while actively suppressing the alternative (Fig. 1j). Notably, only a few activating transcription factors were shared between the two lineages (Fig. 1h), including *GCM1*, whose loss impairs formation of both EVTs and SCTs^22^. Even these shared factors engaged distinct target gene sets in the two cell types (Extended Data Fig. 3g), highlighting extensive regulatory rewiring that secures mutually exclusive trophoblast fates.

## From single cells to tissue architecture

**Fig. 2:**
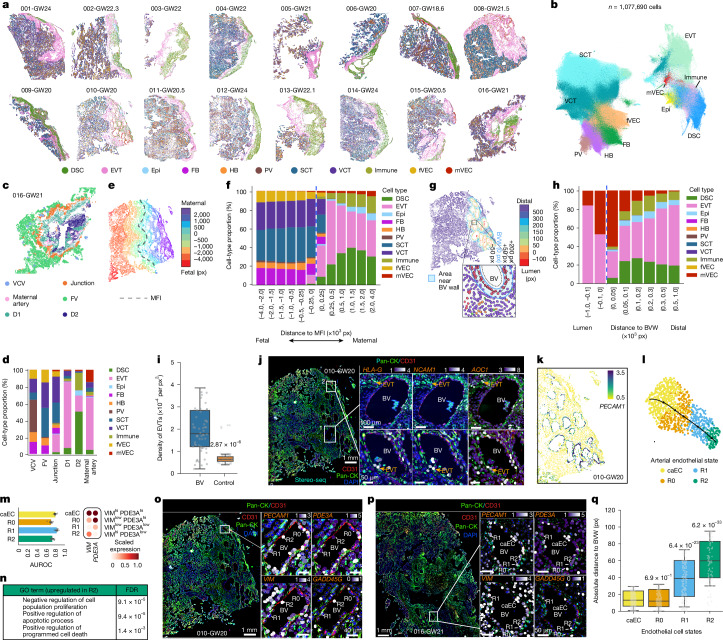
Overview of submicrometre spatial whole-transcriptome profiling at the MFI. **a**, STOmics Stereo-seq profiling of basal-plate sections from 16 second-trimester samples of normal pregnancy. Each dot represents a single cell, colour-coded by cell type. **b**, UMAP projection of around 1.1 million single-cell spatial transcriptomic profiles across 16 samples. **c**, Spatial cell community analysis identified six recurrent communities. D1, decidua-1; D2, decidua-2; FV, floating villus; Junction, maternal–fetal junction; VCV, villous core vessel. **d**, Each spatial community exhibited a characteristic cell-type composition. **e**, Distances (in 0.5-μm pixels) to the MFI for each cell. Positive distances are maternal; negative distances are fetal. px, pixels. **f**, Cell-type proportions binned across the MFI. **g**, Spatial distances (in 0.5-μm pixels) to the nearest blood vessel (BV) wall in the decidua. Positive, intraluminal; negative, extraluminal. **h**, Cell-type proportion in decidua by proximity to blood vessel walls (BVWs). **i**, Quantification of EVT density adjacent to blood vessel walls (*n* = 62) compared to randomized decidual regions of same size. In box plots, the centre line is the median, box represents the IQR, and whiskers show minima and maxima (0.5× IQR) end-points. *P* value, two-tailed Wilcoxon rank-sum test. **j**, Two representative spiral arteries at distinct phases of EVT-mediated remodelling, visualized by joint immunostaining and spatial transcriptomics. Box 1, early stage; box 2, advanced. **k**, Spiral artery cross-sections (*n* = 17) with *PECAM1*^+^ endothelial cells illustrating active EVT invasion in a representative GW20 sample. **l**, Single-cell transcriptomic profiling revealed endothelial state transitions: caEC → R0 → R1 → R2. **m**, *PDE3A* and *VIM* distinguished R0–R2 states with high accuracy (*n* = 10 bootstrap replications). Bars show mean area under the receiver operating characteristic curve (AUROC) and error bars represent s.d. **n**, R2 endothelial cells are enriched for apoptosis-related genes. **o**,**p**, R0–R2 endothelial states in samples 010-GW20 (**o**) and 016-GW21 (**p**) were independently validated via paired spatial transcriptomics and pan-CK and CD31 immunostaining. Fields from one representative sample. **q**, Absolute pixel distances of endothelial states to the vessel walls from 17 remodelling spiral arteries (010-GW20). In box plots, the centre line is the median, box represents the IQR, and whiskers show minima and maxima (0.5× IQR) end-points. *P* values, two-tailed Wilcoxon rank-sum test after Benjamini–Hochberg correction. Source data

**Extended Data Fig. 4:**
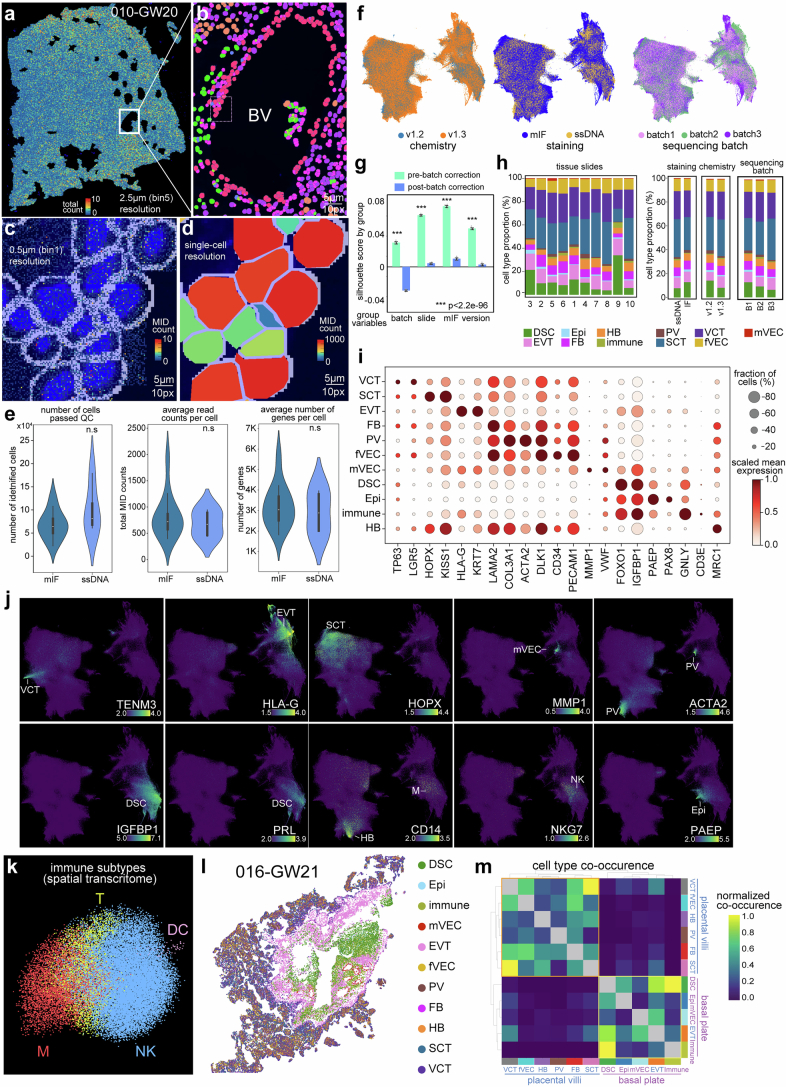
Spatial transcriptomics data quality, integration and clustering. (**a**) Stereo-seq spatial map of a full placental section (010-GW20), with spatial spots colored by total MID counts. (**b**) Zoomed view near a blood vessel (BV) illustrating cell segmentation by Cellpose; each segmented cell (geometric bin) is colored by a cell cluster, with MIDs aggregated within its cell boundary. (**c-d**) Comparison of binning resolutions: (c) raw barcodes at 1-pixel (0.5 µm, bin1) resolution and (d) CellBin-aggregated counts per segmented cell, colored by total MID counts; cell boundaries (white) and nuclei (blue) are shown. (**e**) Comparison of quality metrics between samples processed with mIF or ssDNA staining, showing no significant differences in reconstructed cell numbers, per-cell MID counts, or gene counts. Center line=median, box=IQR (25^th^-75^th^ percentile), minima/maxima=observed minimum and maximum values; n.s., not significant, two-tailed Wilcoxon rank-sum test. (**f**) UMAPs showing consistent cell type identifications across STOmics chemistry versions, staining strategies (mIF vs. ssDNA), and sequencing batches. (**g**) Silhouette scores for technical variables before and after Harmony correction, showing effective batch correction and improved mixing post-correction (Bar plots, mean ± s.d.; ***, P < 0.001; two-tailed Wilcoxon rank-sum test with Benjamini-Hochberg correction). (**h**) Cell type proportions stratified by tissue slide (16 samples on 10 slides), chemistry version, and sequencing batch, indicating minimal technical bias and consistent cell type representation after integration. (**i**) Dot plot of key marker gene expression across major cell types in spatial transcriptomic data. (**j**) UMAPs showing representative marker genes for major cell types (e.g., *TENM3*, VCT; *HLA-G*, EVT; *HOPX*, SCT; *MMP1*, EC; *ACTA2*, PV; *IGFBP1/PRL*, DSC; *CD14*, HB/macrophages; *NKG7*, NK; *PAEP*, epithelium). (**k**) UMAPs of immune cell subtypes identified from spatial transcriptomics (DC, dendritic cells; M, macrophages; NK, natural killer cells; T, T cells). (**l**) High-resolution spatial map of a section (016-GW21), with cells colored by cell types. (**m**) Heatmap of normalized inter-cell-type co-occurrence frequencies within a 300-pixel radius. Source data

To enable spatial mapping of deeply sequenced cell states in a native tissue context, we generated submicrometre (0.5-µm) spatial whole-transcriptomic maps using STOmics Stereo-seq^23,24^ (1 cm × 1 cm chips) for 16 wide-swath basal plate biopsy sections from normal pregnancies (after RNA quality control; Methods), each from an independent donor (Fig. 2a). Stereo-seq enables single-cell segmentation from full-tissue sections and reconstructs single-cell whole transcriptomes by aggregating transcripts captured at 0.5-µm spatial resolution (Extended Data Fig. 4a–d). Focusing on the peak phase of EVT invasion and spiral artery remodelling, we profiled only second-trimester basal plate specimens that contained a well-defined MFI with anchoring villi and abundant spiral arteries (Supplementary Table 4 and Methods). The technology permits immunostaining prior to transcriptomic profiling. Most of the samples (12 out of 16) were immunostained with anti-pan-cytokeratin (CK) to label trophoblasts, and a subset that contained numerous blood vessels was co-stained with anti-CD31 to mark the vasculature. The remaining four samples were deliberately processed without immunostaining to assess its impact on transcriptomic quality and to validate annotations using transcriptomes alone. Extensive quality control confirmed data quality and the manufacturer’s recommendation that pre-run immunostaining did not compromise Stereo-seq performance (Extended Data Fig. 4e and Methods). Cell-type assignments were concordant across technical variations, such as staining protocols and chemistry versions (Extended Data Fig. 4f–h), supporting consistency across preparation methods and justifying integrating all 16 whole-slide samples for downstream analysis (Fig. 2a).

**Extended Data Fig. 5:**
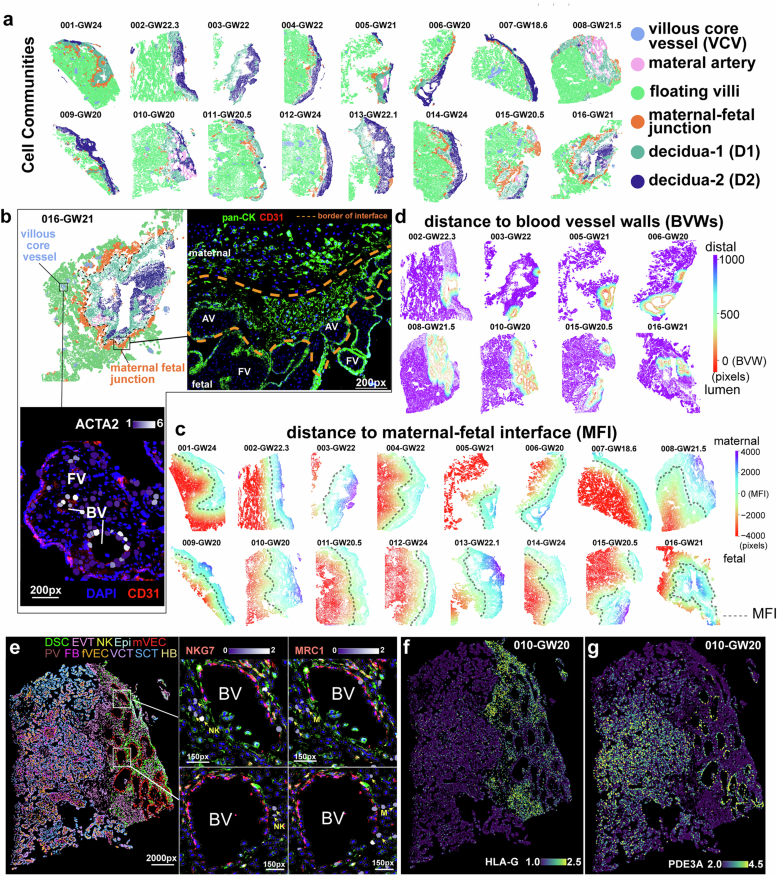
Spatial mapping of cell types. (**a**) Six cell communities identified across 16 samples, with regions annotated as villous core vessels (VCV), maternal artery, floating villi (FV), maternal-fetal junction (Junction), decidua-1 (D1), and decidua-2 (D2). (**b**) Zoomed views of a VCV niche (bottom; *ACTA2*^+^ perivascular cells) and a junction niche (right) at GW21, immunostained for pan-CK (green) and CD31 (red); interface boundary=orange dashed line. AV, anchoring villi; BV, blood vessel. (**c**) Spatial map of distance to the maternal-fetal interface across 16 samples (positive, maternal; negative, fetal). (**d**) Spatial map of distance to maternal spiral artery vessel walls (BVWs) across 8 samples with detectable uterine vessels. Positive values, extraluminal; negative values, intraluminal. (**e**) Spatial views (GW20 basal plate) showing immune cells adjacent to a BV. Arrows indicate NK cells (*NKG7*) and macrophages (*CD14*). (**f-g**) Spatial maps showing aggregation of *HLA-G*^+^ EVTs (**f**) around *PDE3A*^+^ spiral arteries (**g**) undergoing active remodeling.

Our spatial profiling reconstructed around 1.1 million cells across all samples; their transcriptomes were harmonized and embedded in a unified uniform manifold approximation and projection (UMAP) that recapitulated the major cell types (Fig. 2b, Extended Data Fig. 4i–l and Methods). We performed spatial co-occurrence analysis to identify significantly co-localizing cell populations in the decidua, which revealed the strongest associations between decidual stromal and immune cells (Extended Data Fig. 4m), suggesting stromal–immune crosstalk^25^. We next performed spatial cell community analysis to identify fundamental ‘neighbourhoods’, spatial niches of adjoining cells that recur across tissues and share characteristic cell-type compositions^26,27^. This analysis revealed six major communities, each corresponding to a distinct anatomical niche: two decidual niches (D1 and D2), maternal arterial and fetal villous core vascular niches, the maternal–fetal junction, and the floating villi niche (see Fig. 2c for a representative section and Extended Data Fig. 5a for all tissues). Each niche had a characteristic cell-type composition (Fig. 2d).

The decidual niches D1 and D2 had distinct microenvironments. D1 was enriched for EVTs, whereas D2 had fewer EVTs and was dominated by decidual stromal and immune cells (Fig. 2d). The vascular communities also diverged. The maternal arterial niche was enriched for EVTs that were probably engaged in vessel remodelling, whereas the villous core vascular niche was enriched for *ACTA2*^+^ perivascular cells (Extended Data Fig. 5b, bottom). At the maternal–fetal junction, anchoring villus VCTs and SCTs intermingled with decidual stromal and endothelial cells, whereas the floating villi niche comprised SCTs, VCTs and fetal endothelial cells lacking a perivascular sheath (Fig. 2d), consistent with capillary networks beneath the trophoblast basement membrane. Thus, spatial community analysis delineated recurrent cellular niches that constitute fundamental structural units of the human MFI.

## Mosaic EVTs in maternal vessel remodelling

For each cell, we computed the pixel distance (pixel size, 0.5 µm) to two anatomic landmarks: the nearest portion of the MFI and the nearest maternal spiral artery. In each sample, we first defined the interface as the boundary formed by the termini of dense anchoring villi between the placenta and decidua (Extended Data Fig. 5b, right). Cells were then binned by distance to the interface (Fig. 2e for a representative section; Extended Data Fig. 5c for all sections), and distance-stratified cell-type composition profiles were computed across samples (Fig. 2f). Overall, EVTs were more abundant in superficial decidua than in deep decidua, consistent with only the most invasive EVTs reaching those depths. However, immune and DSCs were enriched in deep decidua. The fetal cell-type composition within chorionic villi was less variable, irrespective of their distance to the interface (Fig. 2f).

Across all sections, CD31 immunostaining together with *PECAM1* expression (CD31 is encoded by *PECAM1*) delineated 62 uterine blood vessels. Detailed review confirmed spiral artery morphology and the expected enrichment of adjacent CK^+^ EVTs. Setting the vessel wall as distance zero, we quantified cell-type composition in concentric 100-pixel distance bins (Fig. 2g for a representative section; Extended Data Fig. 5d for all vessel-containing sections), assigning positive distances to extraluminal cells and negative distances to intraluminal cells. Distal cells extending into chorionic villi were excluded from the analysis. Luminal and perivascular regions were enriched for maternal endothelial cells and EVTs, with only a small fraction of immune cells (Fig. 2h). Moving outward, endothelial cells were reduced and EVTs remained; decidual and immune cells were the major adjacent populations.

To quantitatively assess EVT aggregation around blood vessels, we estimated their density relative to randomly sampled size-equivalent decidua areas that lacked uterine blood vessels, revealing strong enrichment of EVTs surrounding uterine vessels (Fig. 2i, Methods and Supplementary Table 4). Magnified views of two representative vessels from the same sample (Fig. 2j) highlight the power of integrated immunostaining and spatial transcriptomics to capture distinct stages of EVT-mediated vascular remodelling. The first spiral artery (Fig. 2j, box 1) displayed a mostly intact CD31^+^ endothelial lining, with sparse CK^+^ EVTs clustered on one side (Fig. 2j), indicating an early stage of remodelling. By contrast, the second vessel (Fig. 2j, box 2) had a mosaic EVT phenotype: *NCAM1*^+^ (endovascular EVT marker^28^; Fig. 2j) EVTs co-expressed varying levels of *HLA-G* (Fig. 2j) and *AOC1* (interstitial EVT marker^29,30^) (Fig. 2j). Residual CD31^+^ endothelial cells were sparse and fragmented along the vessel wall, consistent with EVT-mediated displacement and advanced remodelling. Decidual stromal and scattered immune cells were also present (Extended Data Fig. 5e), suggesting multicellular coordination during vessel remodulation.

## Mapping arterial endothelial states

**Extended Data Fig. 6:**
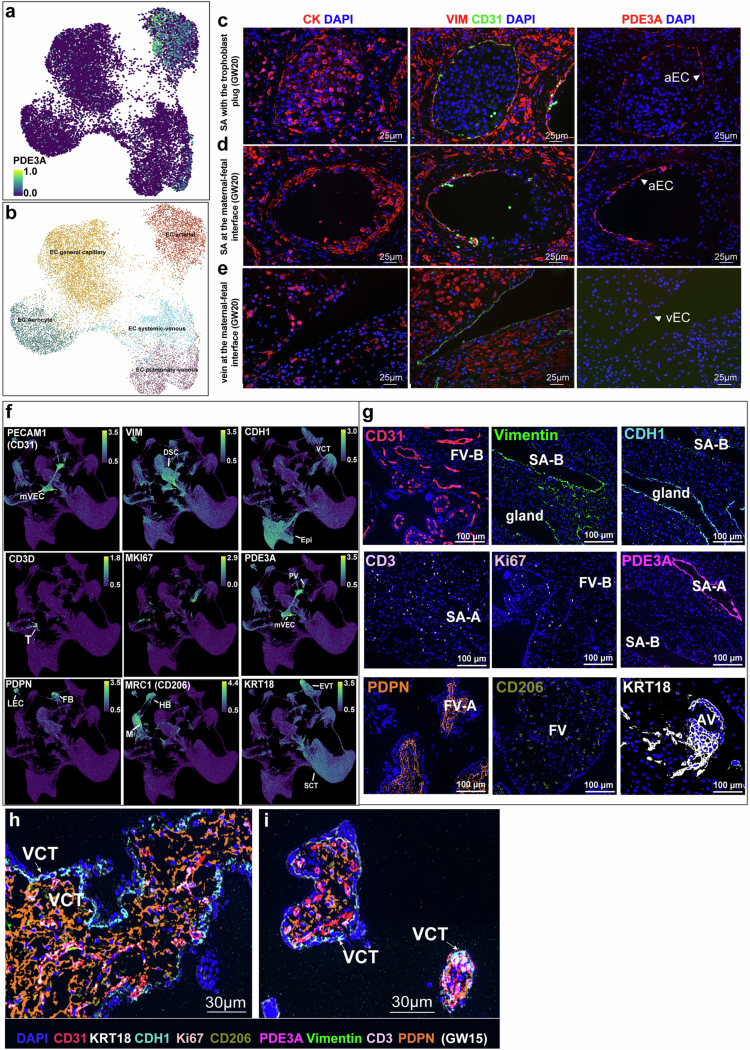
Molecular and spatial validation of arterial endothelial cell markers. (**a-b**) UMAPs showing specific expression of *PDE3A* in arterial endothelial cells (aECs) (a) among all endothelial subtypes in Schupp et al.^31^ (b). (**c-e**) Immunolocalization validating *PDE3A* as an aEC marker in spiral arteries (SA). aECs in SA with (c) or without trophoblast plugs (d) are VIM^+^, CD31^+^, and PDE3A^+^. Trophoblast plugs and partial loss of aECs along vessel wall confirm arterial identity. *PDE3A* is absent from venous ECs (e), which lack dense surrounding CK^+^ trophoblasts. Representative images from one of three biological replicates were shown. (**f**) UMAPs of snRNA-seq data showing specific expression of nine marker genes selected for the CODEX panel. (**g**) Single-channel CODEX images (GW15 basal plate) illustrating spatial localization of each marker: CD3 (T cells), CDH1 (endometrial epithelium and cytotrophoblasts), PDPN (lymphatic endothelium and fetal fibroblasts), Vimentin (decidual stromal cells and subsets of vascular/lymphatic endothelium), CD31 (ECs), CK18 (*KRT18*, cytotrophoblasts), CD206 (macrophages and Hofbauer cells), and PDE3A (aECs and perivascular smooth muscle). SA, spiral artery; FV, floating villi; AV, anchoring villi. (**h–i**) Representative zoomed views showing VCT-specific CDH1 expression (cyan; arrows) in placental villi (GW15 basal plate).

In sample 010-GW20, from a coiled spiral artery, we identified 17 cross-sections that traversed the decidua, and each had varying densities of endothelial cells and EVTs lining the vessel walls (Fig. 2k and Extended Data Fig. 5f,g for endothelial and EVT localization, respectively). This series in a single sample enabled detailed mapping of endothelial state transitions without confounding inter-sample variability. We later replicated these transitions in additional samples and validated them at the protein level. We used *PDE3A*, a recently identified arterial endothelial marker^31^ that we independently validated by reanalysis (Extended Data Fig. 6a,b) and immunostaining of uterine spiral arteries (Extended Data Fig. 6c–e), to confirm that these vessels were lined by arterial endothelium (Extended Data Fig. 5g). Whole-transcriptome profiling of *PECAM1*^+^ arterial endothelial cells within these arteries followed by pseudotime reconstruction resolved four sequential states (Fig. 2l). Differential expression analysis between adjacent states showed that co-expression of *PDE3A* and *VIM* distinguished each cell state (Fig. 2m, Methods and Supplementary Table 5): canonical arterial endothelial cells (caECs; *VIM*^hi^*PDE3A*^hi^), which progressed through R0 (*VIM*^low^*PDE3A*^hi^), R1 (*VIM*^low^*PDE3A*^low^) and R2 (*VIM*^hi^*PDE3A*^low^) (Fig. 2l,m), charting the endothelial response to EVT-mediated remodelling. Functional enrichment analysis confirmed the trajectory direction: genes upregulated in R2 relative to caECs were significantly associated with apoptosis (Fig. 2n and Supplementary Table 5), confirming R2 as the terminal state. The same cell states (R0, R1 and R2) were observed in independent tissue samples (Fig. 2o,p). Of note, the strong expression of the pro-apoptotic gene *GADD45G* was specific to the R2 terminal state (Fig. 2o). Spatial measurements further validated this trajectory (Fig. 2l). We calculated the absolute distance of each endothelial cell from the vessel wall. caECs and R0 cells were proximate, R1 cells were modestly displaced, and R2 cells were most distant (Fig. 2q). Therefore, the loss of caECs during spiral artery remodelling begins with vessel wall displacement in the R1 state, leading to the full detachment, apoptosis and eventual clearance of R2 cells.

**Fig. 3:**
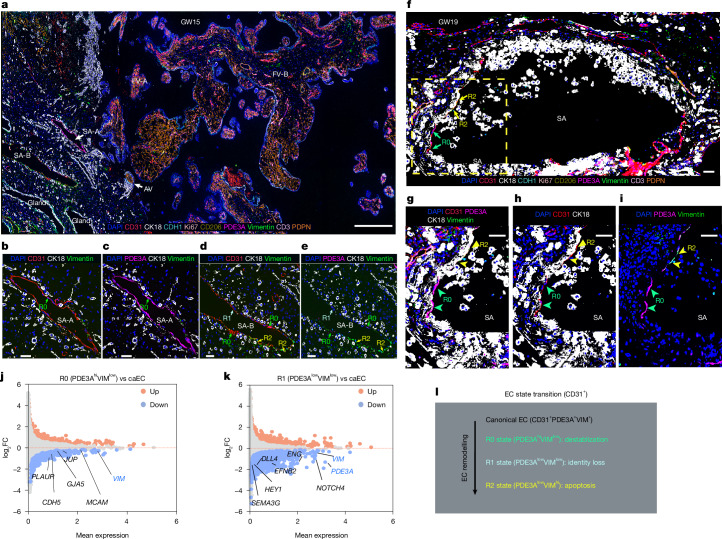
CODEX validation of stepwise arterial endothelial state transitions during EVT remodelling of spiral arteries. **a**, An overview of multiplexed CODEX imaging of a representative tissue section from a human basal plate sample (GW15). The selected markers (and their specificity): DAPI (nuclei), CD31 (endothelial cells), CK18 (cytotrophoblasts, particularly EVTs), CDH1 (endometrial epithelium and cytotrophoblasts), Ki67 (proliferation), CD206 (macrophages and Hofbauer cells), PDE3A (arterial endothelial and perivascular smooth muscle cells), vimentin (decidua stromal cells and a subset of vascular and lymphatic endothelial cells), CD3 (T cells) and PDPN (lymphatic endothelium and fetal fibroblasts). Marker specificities are shown in Extended Data Fig. 6g. SA, spiral artery; AV, anchoring villi. Scale bar, 400 μm. **b**–**e**, Higher-magnification CODEX imaging of spiral arteries SA-A highlighting CD31 (**b**) and PDE3A (**c**), and SA-B highlighting CD31 (**d**) and PDE3A (**e**) recapitulated the transient arterial endothelial states at the protein level: R0 (PDE3A^+^VIM^−^), R1 (PDE3A^−^VIM^−^) and R2 (PDE3A^−^VIM^+^). Biological replicates of the CODEX analysis are included in the Extended Data Fig. 7a. Scale bars, 50 μm. **f**, Overview of CODEX imaging near an extensively remodelled spiral artery from a GW19 basal plate sample. Scale bar, 100 μm. **g**–**i**, Magnification of the boxed region from **f**, showing the details of the distinct endothelial states across selected channels: CD31, PDE3A, CK18 and vimentin (**g**), and CD31 and CK18 (**h**). **i**, R0 (green arrows) and R2 cells (yellow arrows) are spatially segregated. Scale bars, 50 μm. **j**,**k**, Differential gene expression analysis comparing spatial transcriptomic profiles: R0 versus caECs (**j**) and R1 versus caECs (**k**). Genes involved in cell–cell junctions (for example, *PLAUR*, *CDH5* and *JUP*) were down-regulated in R0 versus caECs (**j**), whereas genes that define arterial identity (for example, *DLL4*, *EFNB2*, *HEY1* and *ENG*) were down-regulated in R1 versus caECs (**k**). **l**, Proposed stepwise model of endothelial (CD31^+^) state transitions during spiral artery remodelling. CaECs (*PDE3A*^hi^*VIM*^hi^) → R0 (*PDE3A*^hi^*VIM*^low^) → R1 (*PDE3A*^low^*VIM*^low^) → R2 (*PDE3A*^low^*VIM*^hi^).

**Extended Data Fig. 7:**
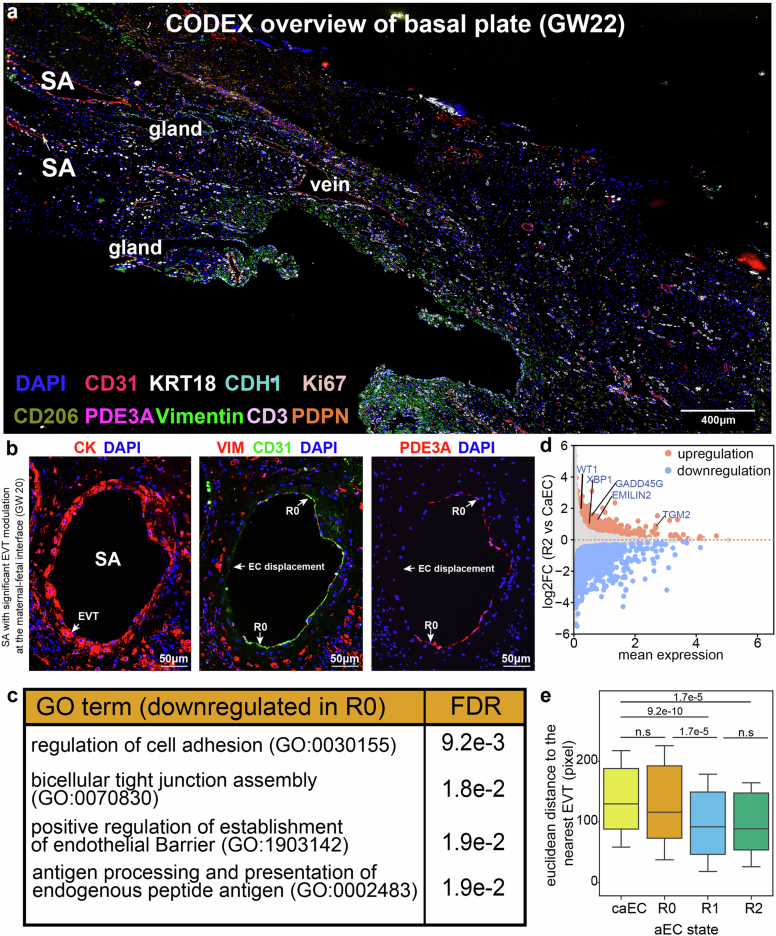
Spatial and functional characterization of the arterial endothelial state transitions. (**a**) Validation of cell type localization at the maternal-fetal interface by CODEX on an independent sample (GW22 basal plate), integrating 9 antibody channels. (**b**) Independent validations using immunolocalization identified one spiral artery (GW 20) undergoing remodeling with discontinuous endothelial lining. Invading EVTs (pan-CK^+^) displace aECs along one side of the vessel (left), while the remaining segment is lined by aECs with distinct molecular states, including R0 (arrows), characterized by VIM^−^ (middle) and PDE3A^+^ (right). (**c**) GO analysis for genes downregulated in R0 aECs vs. canonical aECs. (**d**) Differentially expressed gene analysis in R2 vs. canonical aECs (caECs), showing upregulation of apoptosis-related genes. (**e**) Comparison of spatial proximity to EVTs across aEC states from 17 SAs (010-GW20), quantified as Euclidean distance to the nearest EVT. Box plots, center line=median, box=IQR (25^th^-75^th^ percentile), minima/maxima=lower/upper whisker (0.5×IQR) endpoints, and outliers are not displayed; P values, two-tailed Wilcoxon rank-sum test with Benjamini-Hochberg correction; n.s., not significant. Source data

We next performed CODEX imaging^2^ for protein-level validation by multiplexing nine cell-type-specific antibodies (Supplementary Table 6 and Supplementary Note), which accurately detected the major cell types at the human MFI (Fig. 3a and Extended Data Fig. 6f–i; independent validation in Extended Data Fig. 7a; Methods), including CD31 (*PECAM1*), PDE3A and vimentin (VIM) to label the evolving endothelial cell states. At the protein level, the cell states (R0, R1 and R2) were observed in arteries at different stages of vascular remodelling (SA-A and SA-B at GW15.2 in Fig. 3b–e and a vessel at GW19 in Fig. 3f–i). These CODEX observations were validated by immunolocalization on independent samples (Extended Data Fig. 7b).

For functional characterization, we compared each endothelial state to caECs in the spatial transcriptomes (Fig. 2l). R0 cells exhibited down-regulation of junctional genes (FDR = 1.8 × 10^−2^) and antigen-presentation pathways (FDR = 1.9 × 10^−2^; Fig. 3j, Extended Data Fig. 7c and Supplementary Table 5), defining a vessel wall-adherent ‘primed’ state (Fig. 2q) with reduced structural stability and immune signalling. R1 cells down-regulated *PDE3A* and other arterial markers (for example, *EFNB2*, *HEY1* and *DLL4*; Fig. 3k and Supplementary Table 5), indicating loss of arterial identity, coincident with early detachment from the vessel wall (Fig. 2q). R2, the terminal EVT remodelling state, showed apoptotic gene activation (Fig. 2n and Extended Data Fig. 7d) and complete detachment (Figs. 2q and 3f–i), suggesting anoikis. Notably, caECs were furthest from EVTs, which came progressively closer to R1 and R2 (Extended Data Fig. 7e), supporting a spatial gradient in which increasing EVT proximity drives sequential endothelial transitions from loss of arterial identity (R1) to apoptosis (R2) (Fig. 3l).

In this model (Fig. 3l), SA-A cells (Fig. 3b) were primed for remodelling (R0). SA-B showed more advanced remodelling, with R1 cells along the upper wall and EVT-displaced R2 cells along the lower wall. The artery in Fig. 3d represented a near-terminal stage, with EVTs largely replacing the endothelium.

## Trophoblast developmental trajectories

**Fig. 4:**
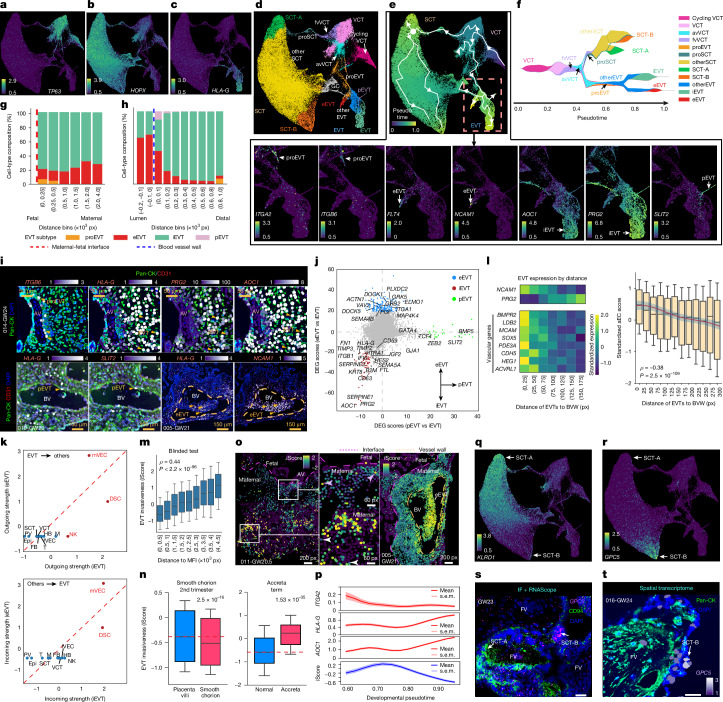
Molecular and spatial characterization of human trophoblast development and function. **a**–**c**, UMAPs of trophoblast subtypes by marker expression: *TP63*^+^ VCTs (**a**), *HOPX*^+^ SCTs (**b**) and *HLA-G*^+^ EVTs (**c**). **d**, Clustering of trophoblast subtypes: iEVT, eEVT, pEVT, giant cell (GC), floating villi VCT (fvVCT), anchoring villi VCT (avVCT), EVT progenitor (proEVT) and SCT progenitor (proSCT). **e**, Reconstructed pseudotime trajectory reveals divergent developmental paths in floating versus anchoring villi by UMAP (top). Bottom, expression of EVT subtype-specific markers on UMAP: proEVT (*ITGA2* and *ITGB6*), eEVT (*FLT4* and *NCAM1*), iEVT (*AOC1* and *PRG2*) and pEVT (*SLIT2*). **f**, Stream plot mapping VCT differentiation. Major branches are shown. **g**,**h**, Proportion of EVT subtypes identified in the decidua binned as the distance from the MFI (**g**) or maternal blood vessel walls (**h**). **i**, Spatial transcriptomics with pan-CK and CD31 immunostaining localized EVT subtypes, which were marked by *HLA-G* versus *ITGB6* (proEVT), *PRG2* and *AOC1* (iEVT), *SLIT2* (pEVT) and *NCAM1* (eEVT). Images highlight EVT positioning near anchoring villi or blood vessels. **j**, Differential expression analysis (pEVT versus iEVT and eEVT versus iEVT) identified subtype-specific signatures. DEG, differentially expressed gene. **k**, CellChat identified distinct outgoing (top) and incoming (bottom) interactions, reciprocal signalling (eEVTs and mVECs), and crosstalk (EVTs and decidual cells). iEVTs showed strong outgoing interactions towards NK cells. **l**, Validation of the EVT pseudovascularization model. Left, EVTs adjacent to vessel walls exhibited high expression of endothelial signatures. Right, positive correlation of endothelial gene scores in EVTs with proximity to vessel walls (*n* = 9,374 EVTs from 16 samples). In box plots, the centre line is the median, box represents the IQR, and whiskers show minima and maxima (0.5× IQR) end-points. *P* value, Spearman (two-tailed) correlation test. **m**, A sparse learning model predicts EVT invasiveness from single-cell transcriptomes. Invasiveness scores (iScores) were positively correlated with EVT decidual depth in the test set (*n* = 63,916 EVTs from 16 samples). In box plots, the centre line is the median, box represent the IQR, and whiskers show minima and maxima (0.5× IQR) end-points. *P* value, Spearman (two-tailed) correlation test. **n**, Benchmark iScores in independent studies. Left, reduced invasiveness of smooth chorion EVTs^40^. Right, EVT shows increased invasiveness in placental accreta spectrum^41^. In box plots, the centre line is the median, box represents the IQR, and whiskers show minima and maxima (0.5× IQR) end-points; outliers are not shown. *P* values, two-tailed Wilcoxon rank-sum test. **o**, Comparison of EVT invasiveness in superficial and deep decidual compartments (left and middle) and near blood vessels (right). **p**, Invasiveness (iScore; bottom) and expression of *ITGA2* (progenitor), *HLA-G* (cell column exit) and *AOC1* (advanced maturation) at EVT maturation stages along the reconstructed trajectories. **q**,**r**, Identification of a novel SCT terminal state with distinct expression of *KLRD1* (encoding CD94) (**q**) and *GPC5* (**r**) in SCT-A versus SCT-B. **s**, Immunolocalization (CD94) and RNAscope (*GPC5*) identifies CD94^+^ SCT-A in syncytium and *GPC5*^+^ SCT-B in syncytial knots of floating villi cells in a representative GW23 sample (*n* = 3). IF, immunofluorescence. Scale bar, 25 μm. **t**, Spatial transcriptomics independently confirmed the presence of the SCT-B subtype in a representative sample (016-GW24, *n* = 16). Immunostaining: pan-CK (green). Scale bar, 25 μm. Source data

**Extended Data Fig. 8:**
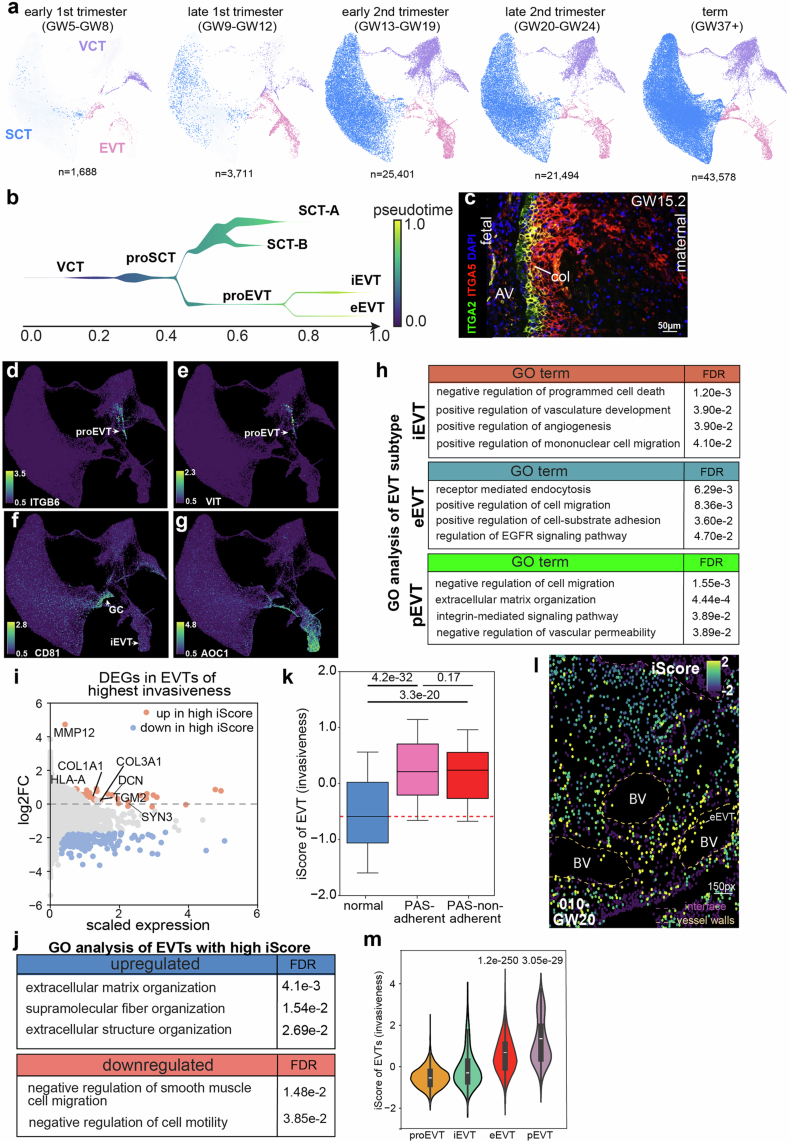
Spatial and functional characterization of EVTs. (**a**) UMAP of all trophoblasts grouped by gestational stages. (**b**) Reconstructed trophoblast differentiation trajectories showing bifurcation from VCT into SCT and EVT lineages. The EVT branch: proEVT, interstitial EVT (iEVT), and endovascular EVT (eEVT), colored by pseudotime. (**c**) Co-immunolocalization validating *ITGA2* expression in EVT progenitors in cell column. Representative fields from one of three biological replicates were shown. (**d-g**) UMAPs showing subtype-specific gene expression in snRNA-seq: *ITGB6* (d) and *VIT* (**e**), proEVT; *AOC1* (**f**), iEVT; and *CD81* (**g**), giant cells (GC). (**h**) Functional enrichment by GO analysis for differentially expressed genes in each EVT subtype. (**i**) Differential expression analysis between highly invasive EVTs (highest 5% iScore) and minimally invasive EVTs (lowest 5%) from the held-out test. (**j**) GO enrichment analysis comparing EVTs with high vs. low iScores. (**k**) Comparison of EVT iScores across normal pregnancy, placenta accreta spectrum (PAS)-adherent, and PAS-nonadherent placentas. Box plots, center line=median, box=IQR (25^th^-75^th^ percentile), minima/maxima=lower/upper whisker (0.5×IQR) endpoints, and outliers were not displayed; P values, two-tailed Wilcoxon rank-sum test with Benjamini-Hochberg correction. (**l**) Spatial distribution of EVT iScores near the maternal-fetal interface and blood vessels (GW20). (**m**) iScore distributions across EVT subtypes (proEVT, iEVT, eEVT, pEVT). Center line=median, box=IQR (25^th^-75^th^ percentile), minima/maxima=observed minimum and maximum values; P values, two-tailed Wilcoxon rank-sum test with Benjamini-Hochberg correction. Source data

Deep-coverage snRNA-seq enabled sensitive identification of intermediate trophoblast states, which were then mapped in situ using spatial transcriptomics. We clustered 95,872 trophoblasts on the basis of snRNA-seq, identifying VCT, SCT and EVT subpopulations distinguished by canonical markers (Fig. 4a–c). Within each lineage, we uncovered multiple intermediate cell states that varied across gestational stages (Fig. 4d and Extended Data Fig. 8a). Pseudotime analysis using Palantir^32^ reconstructed the canonical bifurcation of VCTs into SCTs and EVTs (Fig. 4e) and resolved the timing of emerging trophoblast states along developmental continua (Fig. 4f and Extended Data Fig. 8b). First, we focused on the EVT lineage (Fig. 4e), which originated from a VCT subtype, anchoring villi VCTs (Fig. 4d,f), and was distinct from another subtype, floating villi VCTs, that primarily gave rise to SCTs (Fig. 4d,f). We identified a progenitor EVT population marked by *ITGA2*, *ITGB6* and *VIT* expression^33^ (Fig. 4e and Extended Data Fig. 8c–e), and further resolved three terminal EVT subtypes: endovascular (eEVT), interstitial (iEVT) and perivascular (pEVT) EVTs, each defined by established markers (Fig. 4e). Additionally, we identified a population of potential trophoblast giant cells (Fig. 4d), marked by CD81 (ref. ^3^) (Extended Data Fig. 8f). These cells also expressed canonical iEVT markers (Extended Data Fig. 8g), suggesting their formation from iEVTs; however, their transcriptomes clustered with SCTs (Fig. 4d), probably reflecting shared cell fusion mechanisms.

## Spatial localization of EVT subtypes

We next leveraged the aggregated spatial dataset to localize EVT subtypes as a function of distance from the MFI and vessel wall (Fig. 4g,h). EVT progenitors were confined to regions adjacent to the interface, including an *ITGB6*^+^ subpopulation^33^ that was restricted to cell columns (Fig. 4i). The proportion of eEVTs increased with decidual depth and iEVTs were the predominant subtype across decidual layers (Fig. 4g), initiating *HLA-G* expression as they exited the columns (Fig. 4i). Within iEVTs, *PRG2*^+^ and *AOC1*^+^ subpopulations^29^ (Fig. 4e) were spatially intermingled (Fig. 4i). *AOC1* was enriched in developmentally advanced iEVTs (Fig. 4e), consistent with its role as a late-stage EVT marker^34^. Stratifying by vessel proximity, *NCAM1*^+^ eEVTs were restricted to intraluminal and perivascular zones with concomitant iEVT depletion, whereas *SLIT2*^+^*NCAM1*^−^ pEVTs^35^ (Fig. 4e) were specifically enriched in the perivascular region (Fig. 4h). These observations delineate a mosaic EVT organization in which *HLA-G*^+^ iEVTs encase the vessel, *SLIT2*^+^ pEVTs occupy the perivascular zone, and *NCAM1*^+^ eEVTs populate the lumen and vessel wall (Fig. 4i).

## Functional specializations of EVT subtypes

We compared the transcriptomes of terminally differentiated eEVTs, iEVTs, and pEVTs (Fig. 4j and Supplementary Table 7). Gene ontology analysis highlighted their distinct molecular functions (Extended Data Fig. 8h). CellChat^36^ analyses, which integrates ligand–receptor co-expression with spatial proximity, revealed reciprocal signalling between eEVTs and maternal vascular endothelial cells (mVECs; Fig. 4k). Signals from endothelial cells to eEVTs are likely to recruit eEVTs to spiral arteries, whereas signals from eEVTs to endothelial cells are likely to contribute to the caEC-to-R2 transition (Fig. 3f). This analysis also revealed crosstalk between iEVTs and DSCs (Fig. 4k). Additionally, iEVTs showed strong outgoing signalling towards natural killer (NK) cells, suggesting that iEVTs have a role in modulating NK cells^37^.

## The pseudovascularization model

Previously, we proposed a pseudovascularization model in which eEVT remodelling of spiral arteries entails their mimicry of endothelial phenotypes, supported by their CDH5 expression^38^. Across all spatial transcriptomic samples (Fig. 2a), we validated this model transcriptome-wide. As EVTs approached the vessel wall, they shifted from an interstitial to an endovascular phenotype, down-regulating *PRG2* and up-regulating *NCAM1*. Meanwhile, their expression of endothelial-associated genes peaked in wall-adjacent eEVTs (Fig. 4l, left). Furthermore, an ‘endothelium-like’ score^39^ assigned to each EVT on the basis of the 100 genes that were most enriched in mVECs (from snRNA-seq; Fig. 1b), was significantly inversely correlated with EVT distance from the vessel wall (Fig. 4l, right), suggesting progressive acquisition of an endothelial programme as EVTs occupy the spiral artery niche. Thus, spatially resolved data bolster the pseudovascularization model, showing that eEVTs systematically adopt an endothelial-like identity. In addition, eEVTs co-clustering with other EVT subtypes (from snRNA-seq; Fig. 4d) indicate preserved trophoblast identity alongside endothelial mimicry.

## Machine learning to infer EVT invasiveness

EVTs exhibit heterogeneous invasiveness, prompting us to test whether single-cell transcriptomes predict invasive potential. This analysis was guided by three considerations. First, by focusing on the MFI, deep decidual EVTs were spatially separated from the superficial myometrium, reducing confounding from peri-myometrial EVTs that may have ceased invading. Second, most superficial decidual EVTs (around 77.3%) expressed *AOC1* (Fig. 4i), a marker of mature EVTs^34^, limiting confounding from developmental heterogeneity. Third, because EVT transcriptomes integrate both cell-autonomous and microenvironmental cues, transcriptome-based inference captures both sources of variation relevant to invasion.

We first considered EVTs that were not associated with blood vessels (that is, eEVTs and pEVTs) and trained a sparse learning model to compare EVT transcriptomes at varying decidual depths. The model agnostically selected 54 of 3,192 EVT-enriched genes (from snRNA-seq; Fig. 4e and Supplementary Table 8) and aggregated their expression into an invasiveness score (iScore; Supplementary Note). In the randomly held-out test set (EVTs not used in training), iScores tightly tracked iEVT invasion depth (Fig. 4m), and the up-regulated genes were enriched for migration and extracellular matrix (ECM)-remodelling programmes in high- versus low-scoring cells (Extended Data Fig. 8i,j and Supplementary Note), confirming biological relevance. In independent single-cell datasets, EVTs from the smooth chorion^40^, known to have minimal invasion, indeed showed reduced iScores (Fig. 4n, left). Conversely, EVTs from placenta accreta spectrum pregnancies^41^, characterized by excessive invasion, had higher iScores regardless of whether they were sampled from decidua-adherent or non-adherent regions (Fig. 4n, right and Extended Data Fig. 8k). These independent validations confirmed biological specificity. Across iEVTs from the spatial data, high iScores generally localized to deep decidua, whereas low iScores were superficial (Fig. 4o, left). Outliers included superficial high-iScore iEVTs (ongoing invasion, pink arrows; Fig. 4o, middle) and deep low-iScore iEVTs (attenuated invasion, white arrows; Fig. 4o, middle), suggesting that iScores captured heterogeneous invasive states beyond spatial position alone. Extending to eEVTs, they showed high iScores near vessels (Fig. 4o, right and Extended Data Fig. 8l). Scoring EVTs from our snRNA-seq data, progenitors exhibited the lowest iScores, whereas eEVTs and pEVTs had the highest (Extended Data Fig. 8m). iEVTs had a stage-dependent pattern: iScores rose sharply as *HLA-G*^+^ cells entered the decidua and then declined with *AOC1*^+^ expression and maturation (Fig. 4p). Thus, iEVT invasive potential is inversely correlated with developmental maturation; this antagonism suggests a mechanistic basis for tightly controlled uterine invasion.

## A novel SCT subtype in syncytial knots

**Extended Data Fig. 9:**
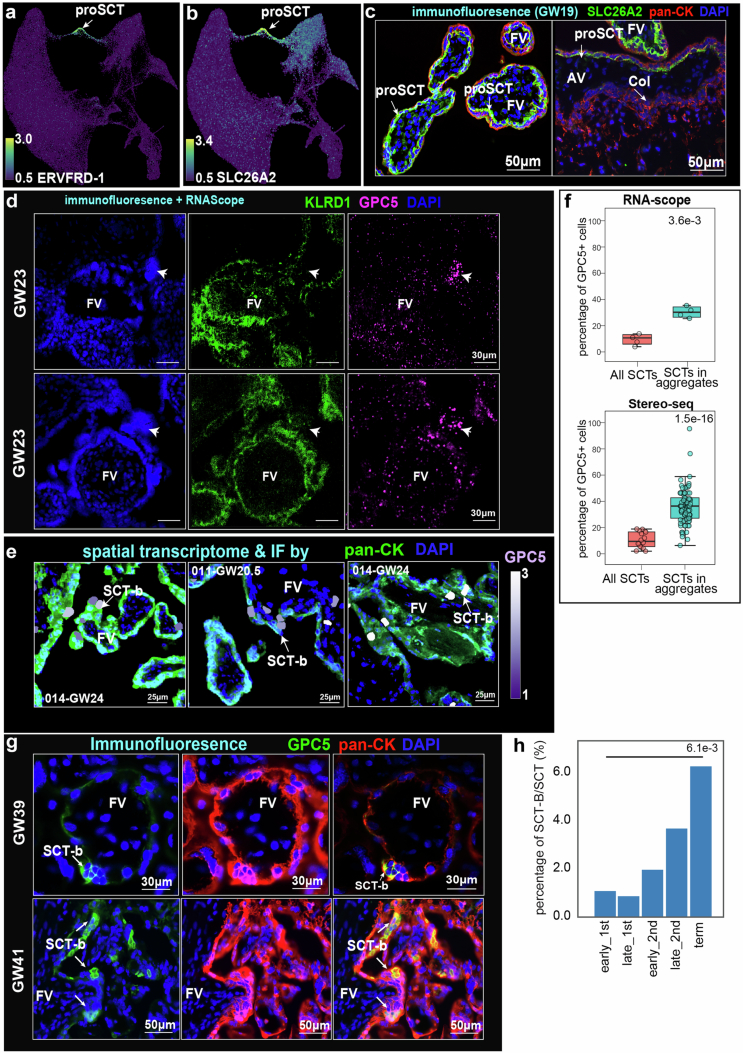
Molecular and spatial characterization of syncytiotrophoblast subtypes. (**a-b**) snRNA-seq UMAPs showing *ERVFRD-1* (syncytin-2) (a) and *SLC26A2* (b) expression specific to progenitor SCTs (proSCT). (**c**) Immunolocalization of *SLC26A2* (green) and pan-CK (red) in placental villi (left) and basal plate (right) marking proSCTs in floating villi. FV, floating villi; AV, anchoring villi; col, cell column (GW19). Representative images from FVs and AVs from one of three replicates were shown. (**d**) Joint RNAscope FISH for *GPC5* (magenta) and immunostaining for CD94 (green) in FV from two GW23 placentas as biological replicates, identifying *GPC5*^+^ SCT-b cells enriched in syncytial aggregates/knots and CD94^+^ SCT-a cells along the trophoblast basement membrane. (**e**) Representative zoomed views from spatial transcriptomes showing *GPC5*^+^ SCT-b localization within syncytial aggregates/knots (011-GW21.5 and 014-GW24), with pan-CK (green) and CD31 (red) co-immunostaining. (**f**) Quantification of *GPC5*^+^ SCTs in syncytial aggregates vs. the entire syncytium by RNAscope (left) and Stereo-seq (right). Box plots, center line=median, box=IQR (25^th^-75^th^ percentile), minima/maxima=lower/upper whisker (0.5×IQR) endpoints; P values, two-tailed Student’s t test. (**g**) Immunolocalization of *GPC5* in two term placentas (GW39, GW41) showing preferential cytoplasmic localization of SCT-b cells within syncytial aggregates/knots. (**h**) Increased proportion of the SCT-b subtype with advancing gestation. P values, two-tailed Fisher’s exact test with Benjamini-Hochberg correction. Source data

Our 10x snRNA-seq data confirmed the previously described SCT progenitor population (proSCT; Fig. 4d), marked by syncytin-2 (*ERVFRD-1*) and *SLC26A2* (refs. ^3,9^) (Extended Data Fig. 9a–c). Immediately downstream, we identified an SCT-A subtype with increased *KLRD1* expression (encoding CD94) (Fig. 4q), and a terminal SCT-B state marked by *GPC5* (Fig. 4r). CD94 immunostaining localized SCT-A to the syncytium of floating villi, whereas RNAscope and spatial transcriptomics showed *GPC5* enrichment in syncytial knots, a finding that was supported by immunolocalization in term villi (Fig. 4s,t, Extended Data Fig. 9d–g and Supplementary Table 9). Consistent with the accumulation of syncytial knots in late gestation^42^, SCT-B nuclei were enriched at later gestational stages (Extended Data Fig. 9h), supporting a trajectory from proSCT to SCT-A and finally to SCT-B in syncytial knots.

## Novel DSC states

**Fig. 5:**
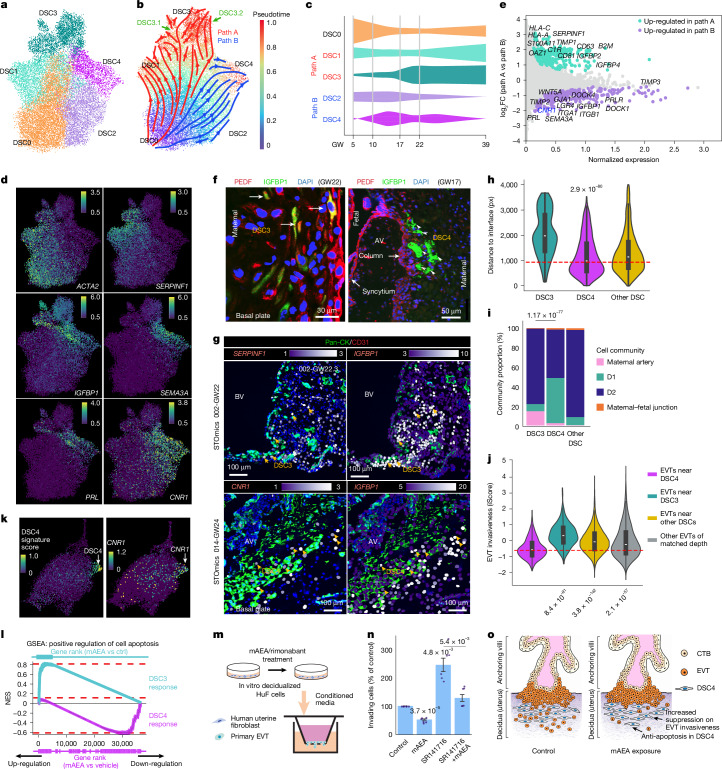
Molecular and spatial characterization of DSC heterogeneity and function. **a**, UMAP projection of five DSC subtypes (DSC0–DSC4) from snRNA-seq. **b**, Reconstructed trajectory of the major decidualization paths: path A (DSC0 → DSC1 → DSC3) and path B (DSC0 → DSC2 → DSC4). **c**, Distribution of DSC subtypes across gestation (GW5–39). **d**, Expression of DSC subtype-specific markers on UMAP: *ACTA2* (undecidualized DSC0), *IGFBP1* and *PRL* (decidualized DSCs, DSC3/DSC4), *SERPINF1* (path A: DSC1/DSC3), *SEMA3A* and *CNR1* (path B: DSC2/DSC4). **e**, Differential expression analysis highlighted *SERPINF1* (encoding PEDF) as a key marker that distinguishes path A from path B DSCs. **f**, Immunolocalization of DSC subtypes: PEDF^+^IGFBP1^+^ DSC3 cells were localized in interstitial decidua (left); PEDF^−^IGFBP1^+^ DSC4 cells were enriched in superficial decidua and clustered near anchoring villi (right). Images from *n* = 3 representative samples. **g**, Spatial transcriptomics: DSC3 cells (*SERPINF1*^+^*PRL*^+^) were adjacent to blood vessels in deep decidua (top); DSC4 cells (*CNR1*^+^*IGFBP1*^+^) were localized near anchoring villi in superficial decidua (bottom). Images from *n* = 16 representative samples. Scale bars, 100 μm. **h**, Spatial distance analysis (*n* = 78,993 DSCs) showed that DSC4 were proximal to the MFI compared with other subtypes (16 samples). In box plots, the centre line is the median, box represents the IQR, and whiskers show observed minimum and maximum values. *P* value, Wilcoxon rank-sum test (two-tailed) after Benjamini–Hochberg correction. **i**, Comparing DSC3 and DSC4 proportions across cell communities. *P* value, chi-square (two-tailed) test after Benjamini–Hochberg correction. **j**, EVTs adjacent to DSC4 exhibited lower iScores than those near DSC3, other DSCs or EVTs at comparable decidual depths (16 samples). In box plots, the centre line is the median, box represents the IQR, and whiskers show observed minimum and maximum values. *P* values, Wilcoxon rank-sum test (two-tailed) after Benjamini–Hochberg correction. **k**, In vitro decidualized HuFs recapitulated the DSC4 transcriptomic signatures (left), manifested by *CNR1* (right). **l**, Gene set enrichment analysis (GSEA) of DSC3 versus DSC4 responses to mAEA after in vitro decidualization. mAEA induces pro-apoptotic genes in DSC3, which were repressed in DSC4. Ctrl, control; NES, normalized enrichment score. **m**, Schematic of Transwell invasion assay. **n**, Quantification of primary cytotrophoblast invasion (*n* = 6). Data are mean ± s.e.m.; *P* values, Student’s *t*-test (two-tailed) after Benjamini–Hochberg correction. **o**, Schematic of model. mAEA exposure protects DSC4 from apoptosis and reshapes DSC4 paracrine cues to enhance local suppression on adjacent EVT invasiveness. CTB, cytotrophoblast. Source data

**Extended Data Fig. 10:**
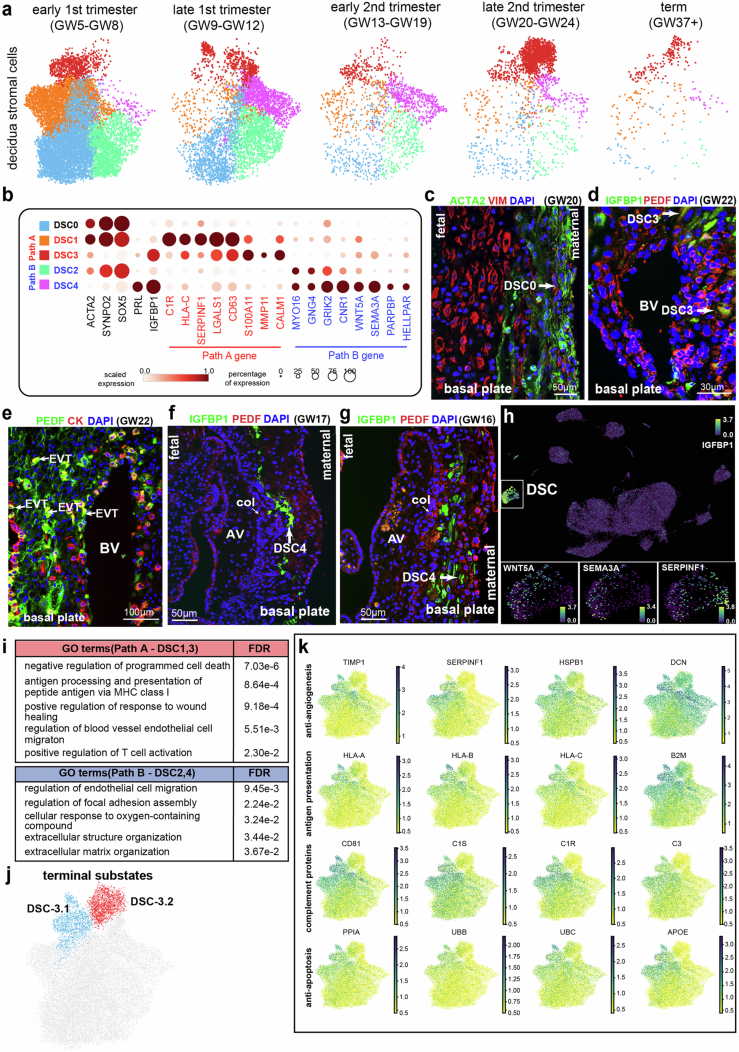
Spatial and functional characterization of decidual stromal cells (DSCs). (**a**) UMAP of DSCs across gestational ages colored by subtypes. (**b**) Dot plot showing scaled expression of representative path A and B markers across five DSC subtypes. (**c-g**) Immunolocalization confirming DSC subtype identity and spatial localization (second trimester basal plate); representative fields from at least three biological replicates were shown. (**c**) Undecidualized cells (ACTA2^+^, red) were localized in the deep decidua (GW20); (**d**) PEDF^+^IGFBP1^+^ DSC3 near blood vessels (PEDF, red; IGFBP1, green) in GW22 decidua; (**e**) A subset of CK^+^ (red) EVTs also displayed PEDF protein abundance (green) in the decidua (GW22); (**f-g**) PEDF^−^ (red) /IGFBP1^+^ (green) DSC4 adjacent to anchoring villi (GW17 and GW16). (**h**) Replication scRNA-seq of 28,626 cells from decidual swabs at term recapitulated DSC subtype signatures. (**i**) Enriched GO terms for genes upregulated in path A and path B DSCs. (**j**) UMAP showing two DSC3 subclusters (3.1 and 3.2) identified in snRNA-seq. (**k**) DSC3.2 displayed significant downregulation of genes in anti-angiogenesis, antigen presentation, complement activation, and anti-apoptosis, relative to DSC3.1 cells.

snRNA-seq of 20,579 DSCs across gestation identified five distinct clusters (DSC0–DSC4; Fig. 5a and Extended Data Fig. 10a). Pseudotime analysis revealed two trajectories rooted in DSC0: path A (DSC0 → DSC1 → DSC3) and path B (DSC0 → DSC2 → DSC4; Fig. 5b). DSC0 predominated in early gestation, whereas DSC3 and DSC4 emerged later (Fig. 5c). A prior early-pregnancy atlas described only path A^5^ (markers below); thus, path B and its terminal DSC4 population represent a previously unrecognized decidual lineage. Notably, DSC4 was also absent from non-pregnant human endometrium (proliferative and secretory phases)^43^, indicating pregnancy-specific emergence.

Marker analysis supported these trajectories. DSC0 cells expressed high levels of *ACTA2* and low *IGFBP1* and *PRL*, indicating an undecidualized state, whereas DSC3 and DSC4 showed low expression of *ACTA2* and strong expression of *IGFBP1*, demonstrating decidualization (Fig. 5d and Extended Data Fig. 10b). *SERPINF1* (encoding PEDF) was specifically up-regulated along path A (Fig. 5d,e), distinguishing DSC3 (PEDF^+^IGFBP1^+^) from DSC4 (PEDF^−^IGFBP1^+^). Immunolocalization identified ACTA2^+^ DSC0 cells deep in the decidua (Extended Data Fig. 10c), whereas DSC3 (PEDF^+^IGFBP1^+^) cells were broadly distributed (Fig. 5f, left), often in perivascular regions (Extended Data Fig. 10d). We also observed a small population of CK^+^ cytotrophoblasts that immunostained for PEDF (Fig. 5f and Extended Data Fig. 10e). By contrast, the novel DSC4 subtype (PEDF^−^IGFBP1^+^) localized to the superficial decidual surface, and many were adjacent to the termini of anchoring villi (Fig. 5f, right and Extended Data Fig. 10f,g for biological replicates), a pattern later confirmed by spatial transcriptomics. Finally, independent validation using single-cell RNA-seq from 13 additional decidual samples (28,626 cells) reproduced the DSC3 and DSC4 subtypes (Extended Data Fig. 10h).

We next functionally characterized the two DSC developmental pathways (Fig. 5e). Genes that were up-regulated along path A were enriched for endothelial cell migration (FDR = 9.31 × 10^−^^3^; Supplementary Table 10), consistent with the frequent perivascular localization of DSC3 (Extended Data Fig. 10d), and many other functions (Extended Data Fig. 10i and Supplementary Table 10). At the terminus of path A (DSC3), we observed bifurcation into two subclusters, DSC3.1 and DSC3.2 (Fig. 5b and Extended Data Fig. 10j), both of which exhibited *IGFBP1* expression. DSC3.2 cells, which emerged mainly after GW22, broadly down-regulated angiogenic inhibitors, MHC class I molecules, complement components and anti-apoptotic genes (Extended Data Fig. 10k). These features support a senescent-like decidual state, consistent with prior reports that senescent DSCs accumulate at later gestation and contribute to parturition^44^.

**Extended Data Fig. 11:**
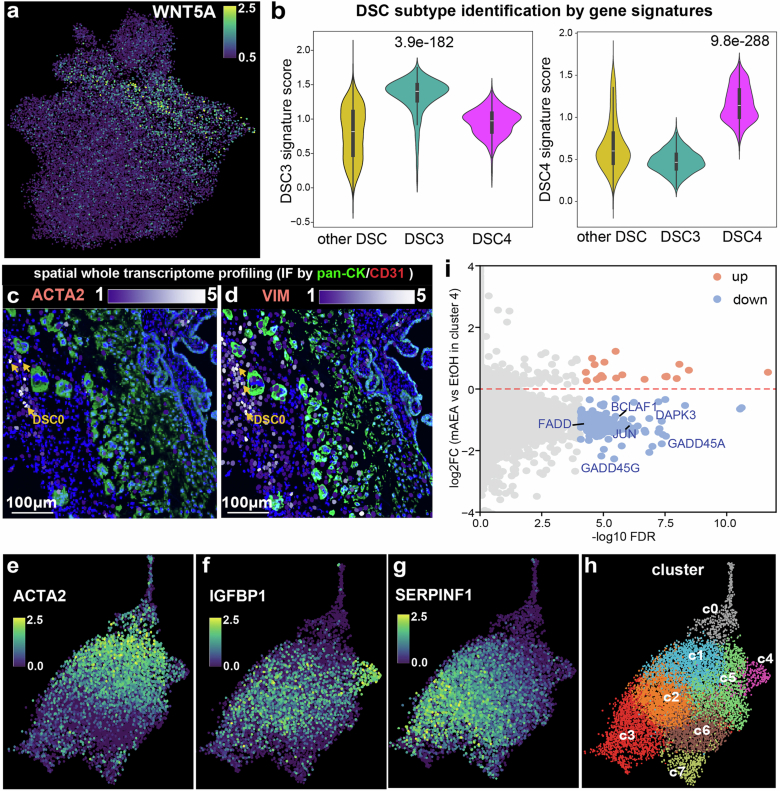
Characterization of DSC4 in in vitro model. (**a**) UMAP showing specific *WNT5A* expression in DSC4. (**b**) Identification of DSC3/DSC4 subtypes from subclusters within the spatial DSC compartment, validated by snRNA-seq signature scoring. Center line=median, box=IQR (25^th^-75^th^ percentile), minima/maxima=observed minimum and maximum values; P values, two-tailed Wilcoxon rank-sum test with Benjamini-Hochberg correction. (**c-d**) Spatial localization of *ACTA2*^+^/*VIM*^+^ undecidualized DSC0 (yellow arrows) overlaid with immunostaining for pan-CK (green) and CD31 (red) in one representative 2^nd^ trimester sample (002-GW22.3, n = 16). (**e-h**) UMAPs of scRNA-seq from in vitro decidualized cells confirming the expected DSC subtypes, marked by *ACTA2* (e, clusters c0, c1 & c5), *IGFBP1* (f, clusters c2–c4), and *SERPINF1* (g, clusters c2 & c3). Clusters c2–c3 resembled DSC3, while cluster c4 resembled DSC4 from primary decidua. (**i**) Differential gene expression showing down-regulation of apoptotic genes in DSC4 upon mAEA exposure. Source data

By contrast, genes that were up-regulated along path B (Fig. 5e) were enriched for ECM organization (FDR = 3.7 × 10^−2^; Supplementary Table 10). DSC4 cells specifically expressed *SEMA3A*, *WNT5A* and *CNR1* (Fig. 5d and Extended Data Fig. 11a). *SEMA3A* and *WNT5A* promote ECM rigidity and stabilization, restraining cell motility^45,46^. The marker *CNR1* encodes the cannabinoid receptor CB1. Because DSC4 cells are in the superficial decidua, often near the termini of anchoring villi (Fig. 5f, right) where EVTs initiate invasion, DSC4 marker expression suggests that DSC4 mediates endocannabinoid signalling at the human MFI and likely serves as a localized brake limiting EVT invasion.

## Regulation of DSC4 on EVT invasion

We first spatially mapped the DSC states and then leveraged the iScore metrics (Fig. 4m) to quantify EVT invasiveness adjacent to DSC4. We used gene signature scores^39^ from snRNA-seq to identify DSC3 and DSC4 subtypes from *IGFBP1*^+^ DSCs on the spatial map (Extended Data Fig. 11b and Methods), whose spatial localizations were consistent with immunostaining (Fig. 5f). *ACTA2*^*+*^*VIM*^*+*^ undecidualized stromal cells (Extended Data Fig. 11c,d) and *SERPINF*^*+*^*IGFBP1*^*+*^ DSC3 cells (Fig. 5g) were enriched in the deep decidua. *CNR1*^*+*^*IGFBP1*^*+*^ DSC4 cells often localized near anchoring villi (Fig. 5g). DSC3 lay farthest from and DSC4 was closest to the MFI (Fig. 5h). When mapped onto spatial niches (Fig. 2c,d), DSC4 were enriched in the EVT-rich D1 niche (Fig. 5i), suggesting interactions between EVT and DSC4. For confirmation, we compared iScores of EVTs adjacent to DSC4 with: (1) EVTs adjacent to other DSC subtypes; and (2) depth-matched EVTs (non-significant depth difference; *P* = 0.85, Wilcoxon rank-sum test). EVTs adjacent to DSC4 had significantly lower iScores than both comparison groups (*P* = 8.4 × 10^−91^ and *P* = 3.8 × 10^−142^, respectively; Wilcoxon rank-sum test; Fig. 5j), revealing a local DSC4-mediated suppression of EVT invasiveness.

## DSC4 cells mediate cannabinoid signalling

Because the cannabinoid receptor CB1 (encoded by *CNR1*) was specifically expressed in DSC4 cells (Fig. 5d,g), and CB1 is activated by the endocannabinoid anandamide (AEA), whose balance at the MFI is critical for pregnancy^47^, we investigated how CB1 agonism affects DSC4 and their regulation of trophoblast invasion. Using our in vitro decidualization model^48^, human uterine fibroblasts (HuFs) were differentiated into decidualized stromal cells and exposed to 0.5 μM methanandamide (mAEA, an AEA analogue) or vehicle for 72 h. Single-cell RNA-seq of 33,088 cells (13,493 control, 19,595 mAEA-exposed; Methods) identified undecidualized *ACTA2*^+^ and decidualized *IGFBP1*^+^ clusters, as well as DSC3- and DSC4-like subtypes as defined by markers of these cell types in primary tissues (Fig. 5k, left and Extended Data Fig. 11e–h). DSC4-like cells retained robust *CNR1* expression (Fig. 5k, right), indicating that the model recapitulated DSC3 and DSC4 differentiation.

Given the absence of *CNR1* in DSC3 and its specific expression in DSC4, we performed subtype-specific differential expression after mAEA exposure (Extended Data Fig. 11i and Supplementary Table 11). Gene set enrichment analysis (Fig. 5l) revealed divergent responses. In DSC3, mAEA tended to up-regulate pro-apoptotic genes (GSEA FDR = 9.7 × 10^−2^), consistent with prior reports of mAEA-induced apoptosis in DSCs^49^. In CB1^+^ DSC4 cells, the apoptosis process was significantly down-regulated (GSEA FDR = 7.0 × 10^−3^), including expression of pro-apoptotic genes (*DAPK3*, *FADD* and *GADD45G*; Extended Data Fig. 11i), suggesting CB1-mediated protection from apoptosis after mAEA exposure.

We tested whether endocannabinoid signalling altered DSC regulation of primary human cytotrophoblasts invasion. Decidualized DSCs were treated for 72 h with mAEA (CB1 agonist), rimonabant (SR141716A, CB1 antagonist), both, or vehicle. After washing, conditioned medium was collected for 24 h and applied to second-trimester human cytotrophoblasts in a Transwell invasion assay (Fig. 5m, Methods and Supplementary Table 12). Conditioned medium from mAEA-treated DSCs reduced cytotrophoblast invasion (*P* = 3.7 × 10^−5^), whereas conditioned medium from rimonabant-treated DSCs increased invasion (*P* = 4.8 × 10^−3^); co-treatment restored invasion towards control (*P* = 5.4 × 10^−3^) (Fig. 5n). Together, these data suggest that endocannabinoid exposure protects DSC4 from apoptosis (Fig. 5l) and modulates DSC4 paracrine cues (Fig. 5n), which enhanced its capacity in constraining adjacent EVT invasiveness (Fig. 5o).

## Cell types that are most vulnerable in disease

**Fig. 6:**
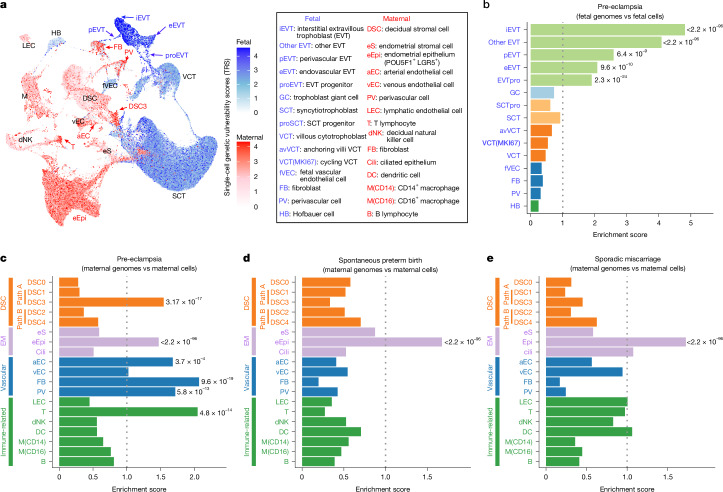
Single-cell risk map of major pregnancy complications. **a**, Single-cell multiomics was integrated with maternal and fetal pre-eclampsia GWASs by mapping high-risk variants onto open chromatin in single cells, revealing cell-type-specific genetic vulnerabilities and pinpointing risk-enriched populations. TRS, trait relevance scores. **b**,**c**, Maternal or fetal cell types and subtypes showing significant associations with pre-eclampsia. The proportion of risk-associated cells within each source was compared to the maternal or fetal background. Enrichment is shown as fold change; *P* values, Fisher’s exact test (two-tailed) with Benjamini–Hochberg correction. **b**, Fetal GWAS risk associations in fetal cell types. **c**, Maternal GWAS risk associations in maternal cell types. EM, endometrium. LEC, lymphatic endothelial cell. **d**,**e**, Parallel enrichment analyses for maternal GWAS risk in spontaneous preterm birth (**d**) and sporadic miscarriage (**e**) across maternal cell types indicate distinct maternal cellular contributors. Only subtypes with more than 200 cells were included. *P* values, Fisher’s exact test (two-tailed) with Benjamini–Hochberg correction. Source data

**Extended Data Fig. 12:**
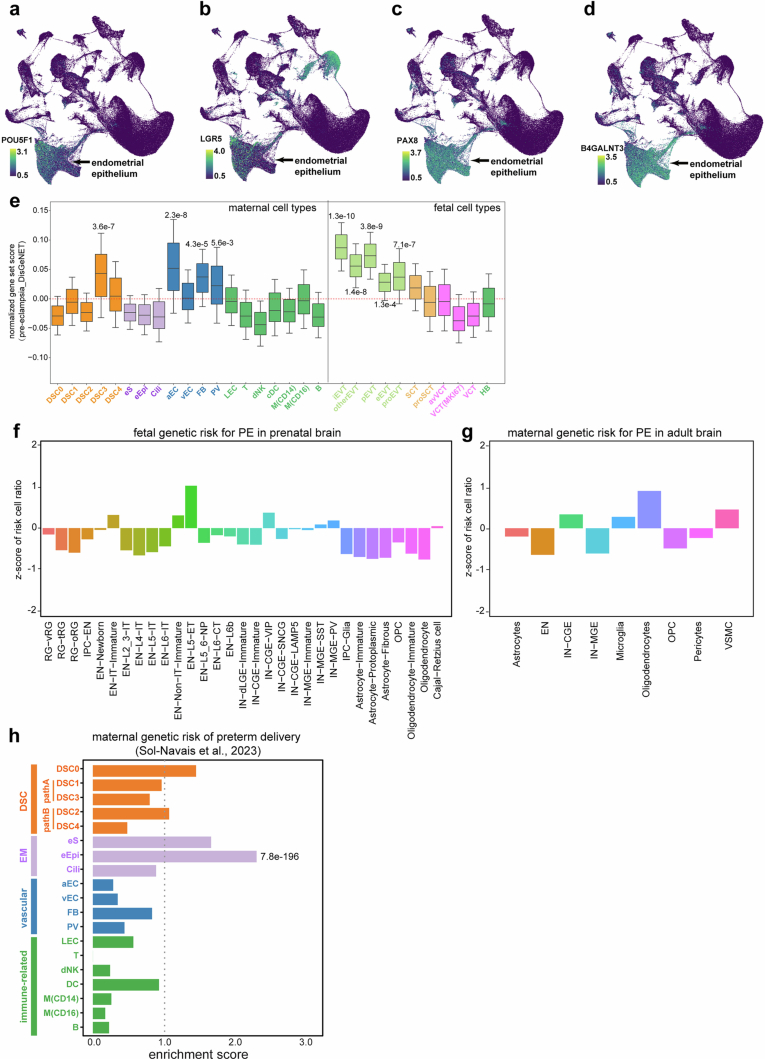
Identify vulnerable cell types in pregnancy complications. (**a-d**) UMAPs showing specific expression of *POU5F1* (OCT4, a), *LGR5* (b), *PAX8* (c), and *B4GALNT3* (d) in endometrial epithelial cells. (**e**) Aggregated preeclampsia gene set scores from DisGeNET across maternal and fetal cell types. Box plots, center line=median, box=IQR (25^th^-75^th^ percentile), minima/maxima=lower/upper whisker (0.5×IQR) endpoints, and outliers are not shown; P values, two-tailed Wilcoxon rank-sum test with Benjamini-Hochberg correction. (**f-g**) Negative controls when performing SCAVENGE analysis in disease irrelevant tissues; no significant preeclampsia risk enrichment in prenatal (**f**) or adult (**g**) neuronal cells; (**h**) Enrichment of maternal GWAS risk for preterm delivery from an independent replication cohort^53^; P values, two-tailed Fisher’s exact test after Benjamini-Hochberg correction. Source data

We next investigated whether our single-cell atlas could explain the genetic architecture of major pregnancy complications. We used SCAVENGE^50^ to integrate single-cell open-chromatin architecture and large-scale (*n* > 10,000) maternal and fetal genome-wide association studies (GWASs) for pre-eclampsia^51^, spontaneous preterm birth^52,53^ and sporadic miscarriage^54^, generating cell-type-resolved maps of genetic risk for each condition (Fig. 6a and Supplementary Table 13). Conventional pre-eclampsia GWAS, despite large cohorts, yielded only a few loci with modest effect sizes, leaving most heritability unexplained^51^. By contrast, SCAVENGE^50^ aggregated GWAS signals across open-chromatin regions in each cell into a per-cell trait relevance score. Pairing maternal cells with maternal pre-eclampsia GWAS^51^ and fetal cells with fetal pre-eclampsia GWAS^51^, we identified 6,221 maternal and 8,232 fetal cells with significant pre-eclampsia risk enrichment. In the fetal compartment, only EVTs, particularly iEVTs, were enriched for pre-eclampsia risk (FDR ≤ 2.2 × 10^−96^) (Fig. 6b). On the maternal side, enriched populations included the DSC3 subtype, arterial endothelium (not venous), perivascular cell, fibroblasts and T cells, whereas decidual macrophages and NK cells were not enriched (Fig. 6c). A distinct *POU5F1*^+^*LGR5*^+^ endometrial epithelial population, restricted to early gestation and expressing stem cell markers (Extended Data Fig. 12a–d), also showed strong enrichment, implicating early endometrial defects in pre-eclampsia. Validation using 151 DisGeNET^55^-curated pre-eclampsia genes confirmed expression enrichment in these vulnerable cell types (Extended Data Fig. 12e and Supplementary Table 13). Negative control experiments paired the same GWASs with fetal brain^56^ or adult brain^57^ single-cell datasets and detected no significant enrichment (Extended Data Fig. 12f,g), demonstrating specificity. Note that this analysis focused on population genetic risk (common variants), and other cell types may contribute through rare or somatic variants or non-genetic factors.

We next applied the same strategy to maternal GWASs for spontaneous preterm birth and sporadic miscarriage, pairing analysis to maternal cell types^52,54^. For both conditions, only *POU5F1*^+^*LGR5*^+^ endometrial epithelial cells showed significant enrichment (Fig. 6d,e), an association that was replicated in an independent spontaneous preterm birth cohort of 233,290 women^53^ (Extended Data Fig. 12h). The shared vulnerability of this epithelial population across pre-eclampsia, spontaneous preterm birth and miscarriage supports the concept of ‘endometrium spectrum disorders’^58^.

## Discussion

Across gestation, our multimodal atlas systematically delineated key cell states, spatial niches, and developmental trajectories at the human MFI and pinpointed genetically vulnerable cell types in major pregnancy complications. In trophoblasts, our study suggested a bistable regulatory circuit that directs commitment to terminal fates from progenitor VCTs (Fig. 1j). Thus, perturbing key regulators could shift this circuit across the EVT–SCT boundary, triggering an aberrant fate switch that may predispose to pathology. A key pathological manifestation of trophoblast dysfunction is dysregulated EVT invasion. This study developed a quantitative framework to quantify EVT invasiveness directly from transcriptomes, with proof-of-concept validation in placenta accreta spectrum cases. While appropriate EVT invasiveness is essential for normal spiral artery remodelling, endothelial cell–intrinsic changes within the vessel wall are also likely to be critical. Our data uncovered discrete endothelial state transitions during vascular remodelling^59^, suggesting that disruption of these short-lived states could impair this process and contribute to pathological changes. The same is likely to extend to other perivascular populations, particularly the decidual stromal subtype DSC3, which often surrounds vessels (Extended Data Fig. 10d) and is genetically associated with pre-eclampsia (Fig. 6c). Its potential role in regulating vascular function warrants future investigation. Together, from a single-cell perspective, coordinated perturbations of trophoblast, endothelial and stromal states are likely to collectively drive many pregnancy complications, shifting the focus from fetal versus maternal contributions to failed integration at the MFI. Although immune cells were not the primary focus of this study, their contribution was evident and will be important to dissect in future work.

We also identified novel cell subtypes and cellular interactions. DSC4, first described here, modulates EVT invasion of the superficial decidua via endocannabinoid signalling. This finding raises public health concerns about cannabis use during pregnancy, as its principal ingredient, Δ9-tetrahydrocannabinol, is a CB1 agonist that is likel to perturb DSC4-mediated endocannabinoid signalling and thereby dysregulate EVT invasion. Consistent with this possibility, prenatal cannabis exposure has been epidemiologically associated with spontaneous preterm birth, stillbirth, reduced birthweight and other adverse outcomes^60^.

Integrating GWASs with our single-cell datasets showed that fetal EVTs strongly mediate population genetic risk of pre-eclampsia. Meanwhile, the *POU5F1*^+^*LGR5*^+^ endometrial epithelial cells exhibited shared genetic risk enrichment across pre-eclampsia, spontaneous preterm birth and miscarriage, highlighting a convergent and previously underappreciated cell population for future mechanistic investigation.

Despite extensive profiling in this study, many more cell states and subtypes almost certainly remain to be discovered, including populations in the deep decidua, at very early gestation and in pregnancy complications. Further work expanding spatial and temporal coverage will be essential to define these elusive populations and to clarify their roles in normal pregnancy and pathological deviations.

## Methods

### Tissue acquisition and processing for joint single-nucleus multiome profiling and spatial transcriptomics

Snap-frozen decidual and basal plate samples were obtained from the existing placenta tissue banks at Stanford University and University of California, San Francisco (Supplementary Tables 1 and 4). All samples were collected with written informed consent. Tissues were derived from women undergoing elective termination of presumed normal pregnancies (first and second-trimester samples; no known or predicted fetal chromosomal abnormalities, Extended Data Fig. 1c) or after term delivery (≥37 gestational weeks). For term samples, clinical records were reviewed to exclude placenta-associated complications (for example, chorioamnionitis); cases with NICU admission and preterm premature rupture of membranes were also excluded. Fresh placental tissues were grossly inspected and dissected under a microscope (Leica Microsystems) by pathologists. Decidua basalis was micro-dissected on ice from the MFI and distinguished from decidua parietalis and capsularis on the basis of characteristic histological and morphological features (Extended Data Fig. 1a,b). Dissected tissues were sequentially washed to remove residual blood cells in DMEM/H-21 medium, supplemented with 12.5% FBS (Hyclone), 1% L-glutamine (Atlanta Biologicals), 1% penicillin/streptomycin and 0.1% gentamicin and cold 1× PBS (Gibco, Thermofisher). Samples used for single-cell or spatial transcriptomic profiling were flash-frozen in liquid nitrogen and stored at −80 °C until processing. RNA quality was assessed from adjacent cryosections using a Bioanalyzer or Tapestation. For fresh frozen samples, only samples with RIN ≥ 7.0 were included.

### Isolation of single nucleus from snap-frozen tissues

Single nuclei were isolated from snap-frozen tissues as previously described^61^ with modification. In brief, tissues were ground on dry ice, and 30–50 mg was homogenized into a pre-chilled 7 ml PYREX dounce homogenizer (Corning Life Science). Tissue was homogenized in 2 ml ice-cold buffer (250 mM sucrose, 0.3% NP-40, 5 mM MgCl_2_, 25 mM KCl, 10 mM Tris-HCl pH 7.8) supplemented with protease inhibitors (Roche, cOmplete) and 0.6 U µl^−1^ Ribolock RNase inhibitor (thermofisher). Debris was removed by 40 μm filtration, and nuclei were purified by OptiPrep iodixanol gradient centrifugation (25%, 30%, 40%). After centrifugation in a swinging bucket centrifuge (Eppendorf 5810R) for 30 min at 3,000*g*, nuclei were collected from the 30–40% interface, washed, and assessed by trypan blue staining and microscopy to ensure nuclei integrity. Approximately 15,000 nuclei per sample were processed using the Chromium Next GEM Single Cell Multiome ATAC + Gene Expression platform (10x Genomics).

### Tissue preparation and CODEX imaging

Placenta samples for CODEX were OCT-embedded, cryosectioned at 10 µm, mounted on poly-L-lysine-coated slides and stored at −80 °C. On the day of staining, sections were equilibrated, acetone-treated, rehydrated, fixed with 1.6% paraformaldehyde, blocked, and incubated with a barcoded antibody cocktail (200 µl/section) for 3 h at room temperature. Sections were then washed, post-fixed with 4% paraformaldehyde and cold methanol, stabilized using CODEX fixative reagent, and stored in storage buffer at 4 °C (≤2 weeks) before imaging. Multiplex imaging was performed on an Akoya CODEX microfluidic system coupled to an inverted fluorescence microscope using a 7-cycle protocol (including blank cycles for alignment) across 4 channels (DAPI, FITC, Cy3 and Cy5) with a 20×/0.75 NA objective. Images were processed using CODEX Analysis Manager. The antibody panel included Akoya-validated barcoded antibodies and custom-conjugated antibodies generated using Akoya oligo barcodes following the manufacturer’s protocol (Supplementary Table 6). Additional details are provided in the Supplementary Note.

### Single-nucleus multiome library construction and sequencing

Single-nucleus Multiome libraries were prepared using the Chromium Next GEM Single Cell Multiome ATAC + Gene Expression kit (10x Genomics) following the manufacturer’s protocol, using one reagent kit per sample. Around 15,000 isolated nuclei per sample were encapsulated into Gel Bead-In Emulsions (GEMs) containing unique cell barcodes, where reverse transcription and transposition occurred, followed by library amplification, and separation of gene expression and chromatin accessibility libraries. Libraries were sequenced on an Illumina NovaSeq 6000 using paired-end reads, with sequencing depth selected on the basis of recommendations from 10x Genomics. On average, each sample yielded approximately 250 million paired-end reads for ATAC and RNA libraries. Raw BCL files were demultiplexed, aligned to the GRCh38 (v.3.0.0) reference genome, and processed for barcode assignment, UMI counting, and quality control using Cell Ranger ARC v.2.0.0 (10x Genomics) (https://support.10xgenomics.com/single-cell-geneexpression/software/pipelines/latest/advanced/references).

### Single-nucleus multiome data processing

High-quality nuclei with paired snRNA-seq and snATAC–seq profiles were retained using the following criteria: RNA UMI counts 1,000–50,000; detected genes >400; mitochondrial reads <20%; ATAC fragment counts 1,000–100,000; transcription start site enrichment >1.0; and nucleosome signal <2.0. Doublets were identified and removed using Scrublet^62^ with prior set to 0.1. After filtering, 191,735 nuclei were retained with paired snATAC- and snRNA-seq data. For snATAC–seq, open-chromatin peaks were called per sample using MACS2 (v.2.2.7)^63^, and merged into a unified peak set after excluding ENCODE blacklist regions^64^. Peak-by-cell count matrices were integrated across samples using reciprocal latent semantic indexing (LSI) projection in Signac^12^. For snRNA-seq, gene expression count matrices were integrated using reciprocal principal components analysis projection in Seurat (v.4)^39^. Prior to integration, data were normalized, scaled and feature-selected following best practices recommended in Seurat/Signac workflows^12^.

Dimensionality reduction was performed using principal components analysis on the top 3,000 variable genes (RNA) and LSI on the top variable ATAC peaks observed in at least 10 cells. UMAP embeddings for RNA/ATAC spaces were projected individually for visualization. Clustering was performed using the Louvain algorithm in Seurat/Signac^12,39^, and clusters were annotated on the basis of canonical marker genes (Supplementary Table 2) using gene expression and gene activity scores. Concordance between RNA- and ATAC-based clustering was assessed using the Jaccard index and percentage of cells assigned to same cell type.

Cell-of-origin assignment was performed using Souporcell^10^ with default parameters. Variants were called using FreeBayes based on 1000 Genomes Project reference, and only informative heterozygous loci (≥5 cells with both reference and alternative alleles) were retained. Trophoblasts were used as fetal reference populations, whereas lymphatic endothelial cells and DSCs were used as maternal reference populations to inform Souporcell assignment. Assignment accuracy was validated using *UTY* expression in samples from male fetuses, in which 94.3% of *UTY*^+^ nuclei were correctly classified as fetal.

### Transcriptional regulation and ATAC–seq footprinting analysis

Putative enhancer peaks were identified by intersecting ATAC–seq fragments with experimentally validated enhancers from FANTOM5 datasets^13^. An enhancer-by-cell accessibility matrix was constructed by counting overlapping fragments per cell. Cell-type–specific enhancers were identified using a log-ratio test, comparing chromatin accessibility in each cell type to all others; enhancers with enriched accessibility (FDR < 0.05) were considered cell-type-specific.

Transcription factor activity was inferred using chromVAR^11^, which quantifies motif-associated accessibility deviations while correcting for GC content. Motif annotations were obtained from the JASPAR2020 database^65^. Cell-type-enriched transcription factors were those with significantly elevated chromVAR deviation scores in each cell type, identified by Wilcoxon rank-sum test. To validate transcription factor activities at the DNA level, transcription factor footprinting was analysed using Signac^12^. For each candidate transcription factor, footprinting profiles were generated over corresponding binding motif(s) within accessible chromatin regions after correcting for Tn5 insertion bias.

### Gene regulatory network construction

Cell type-specific GRNs were constructed using the CellOracle workflow^15^, integrating chromatin accessibility and gene expression data to model transcription factor–target gene interactions in a lineage-resolved manner. A base GRN scaffold was first constructed from snATAC–seq data via scanning transcription factor-binding motifs within proximal promoters and distal regulatory elements using GimmeMotifs (v.5.0)^66^. Proximal elements were annotated with HOMER^67^, and distal regulatory interactions were inferred on the basis of Cicero^68^ co-accessibility scores to link distal enhancers to putative gene targets. Using this scaffold, cell type-specific GRNs were inferred from snRNA-seq data via regularized Ridge regression implemented in CellOracle. Only statistically significant transcription factor–target gene interactions were retained. Among all transcription factors in the GRNs, DEG analysis identified 71 and 30 transcription factors significantly up-regulated (FDR < 0.01) in EVTs and SCTs, respectively, relative to VCTs. Regulatory interaction strength was evaluated by the absolute model-derived coefficients. Interactions with absolute coefficients exceeding 0.1 were visualized using Cytoscape^69^, and all identified regulatory interactions were included in our downstream analyses.

To identify lineage-specific regulatory rewiring, transcription factor–target gene pairs were classified into four categories: (1) EVT-specific activation (*n* = 224); (2) EVT-specific repression (*n* = 18); (3) SCT-specific activation (*n* = 234); and (4) SCT-specific repression (*n* = 48). Expression distributions of target genes in each category were compared with genome-wide background expression in EVT or SCT populations. For transcription factors up-regulated in both lineages, overlap between their target gene sets was quantified using the Jaccard index to assess shared regulatory programmes. Statistical comparisons were performed using the two-tailed Wilcoxon rank-sum test with Benjamini–Hochberg correction.

### DEG and gene ontology analysis

DEGs between cell types or subtypes were compared using Wilcoxon rank-sums test by SCANPY^70^. A composite DEG score was derived for each gene that summarizes fold changes and the differences in the percentage of expressing cells between two cell types. DEGs were determined by FDR (FDR < 0.05). All non-ribosomal DEGs receiving the highest DEG scores were selected as signature genes to define: (1) aEC states (|DEG score| > 10); (2) eEVT, iEVT and pEVT (|DEG score| > 20); and (3) path A DSC and path B DSC (|DEG score|>20). These top ranked DEGs were included for the downstream gene ontology (GO) analysis using Enrichr^71^, and gene ontology terms with an FDR < 0.05 were considered statistically significant.

### Pseudotime trajectory inference

For pseudotime analysis within arterial endothelial cells (spatial transcriptomics), trophoblasts (snRNA-seq) and DSCs (snRNA-seq), the initial and terminal cell states during cell differentiation were inferred by CellRank (v.1.5.1)^72^. Inferred initial states were selected as the developmental origin for pseudotime analysis by Palantir (v.1.1.0)^32^ with default settings. The fate probability to each identified terminal state was estimated for subsequent visualization. The major developmental branches identified by Palantir were visualized on UMAP projection. For trophoblast analysis, major branches with more than 500 cells were aggregated for stream plot.

### Spatial transcriptomic library preparation and sequencing

The Stereo-seq (STOmics) spatial whole-transcriptome sequencing platform was applied to construct a high-resolution spatial transcriptomic atlas of the human MFI. Stereo-seq captures polyadenylated mRNA directly from tissue sections using spatially barcoded DNA nanoball (DNB) arrays, achieving subcellular resolution (~0.5 µm) with an effective spot diameter of ~220 nm. 16 basal plate samples (GW20–24) were embedded in Tissue-Tek OCT compound (4583, Sakura Finetek) and stored at −80 °C. The anatomical structure of cryosections was visually confirmed under a microscope by a pathologist on the day of the experiments. 10 µm cryosections were dissected using a Leica cryostat and mounted onto Stereo-seq transcriptomics T chips (Complete Genomics). Stereo-seq library preparation was performed per the manufacturer’s guidance (Supplementary Note). In brief, tissue sections were methanol-fixed and subjected to antibody or single stranded DNA (ssDNA) staining prior to tissue permeabilization. Released RNA was reverse-transcribed in situ, followed by tissue removal, cDNA amplification and SPRISelect bead purification. Libraries were sequenced on the DNBSEQ T7 platform (50 bp read 1, 100 bp read 2).

### Spatial transcriptomic data analysis

Raw FASTQ files were processed using the STOmics SAW v.8.1 pipeline. Spatial barcodes (CIDs) were aligned to the chip coordinate grid, and high-quality reads were mapped to the human genome (GRCh38) using STAR^73^. Reads with identical CID–UMI pairs were collapsed to generate the final spatial gene expression matrix at bin1 resolution (0.5 nm × 0.5 nm). DAPI and ssDNA images were used to delineate cell boundaries using deep learning–based segmentation pipeline in the SAW workflow. To interrogate gene expression at single-cell resolution, read counts from all bins within a single cell’s boundary were aggregated to achieve CellBin resolution. Cells with low complexity (n_genes <100), excessive transcript counts (>10,000 MIDs or genes), or high mitochondrial gene content (>20%) were excluded. The count matrices in cells passing quality control were processed and normalized using Stereopy (v.1.6.1)^27^, in which the top 3,000 variable genes were selected for principal components analysis-based dimensionality reduction. Batch effects across donors were corrected by Harmony before UMAP visualization and clustering analysis^74^. Clusters were annotated on the basis of known cell-type- and subtype-specific gene signatures from snRNA-seq or known markers, and were then mapped to spatial coordinates for localization validation. Cell community and neighbourhood analysis were performed using Stereopy (v.1.6.1)^27^. Integrated visualization of cell segmentation, gene expression, and immunofluorescence signals was performed on StereoMap (v.4.1). Detailed experimental protocol and downstream spatial data analysis are described in Supplementary Note.

### Spatially resolved cell–cell communication analysis

Intercellular signalling among spatially resolved cell types was analysed using CellChat (v.2)^36^. Analyses were performed with the CellChatDB (the human ligand–receptor database), following the standard workflow. Spatial coordinates were incorporated to constrain inferred intercellular interactions within a maximum Euclidean distance of 200 µm, consistent with the expected physical range of paracrine signalling. Ligand–receptor pairs were grouped into signalling pathways, and communication probabilities were aggregated to estimate pathway-level and overall interaction strengths between cell-type pairs. Communication scores were standardized for comparison. Statistical significance was assessed by label-shuffled permutation (*n* = 1,000) and interactions with Benjamini–Hochberg corrected *P* < 0.05 were considered significant.

### Model-based prediction of EVT invasiveness

Gene signatures defining EVT invasiveness potential were identified via a supervised machine learning framework using L1-regularized regression (LASSO) based on spatial transcriptomic profiles. All identified EVTs excluding those associated with spiral arteries from sixteen sections were split into training and testing datasets at a 1:1 ratio. EVTs were ordered by their distance to the MFI. We then grouped ten cells with similar MFI distances within a narrow depth window to generate pseudobulk profiles, thereby matching spatial context across samples and reducing sparsity and batch effects during model training. A LASSO regression model was trained on the averaged gene expression as predictors and distance to the MFI as the response variable. The feature space comprised 3,192 genes enriched in EVTs relative to other cell types from the independent snRNA-seq data and analysis. Gene expression from training and test datasets were independently standardized while preventing information leakage from the training data into the test data. The model agnostically identified 54 predicting EVT invasiveness potential, with nonzero coefficients, where positive and negative coefficients indicated pro-invasive and anti-invasive effects, respectively. The trained model was applied to single-cell data to generate predicted invasion scores (iScores). Model performance was evaluated by Spearman correlation between predicted iScores and observed distance of each EVT (the held-out test dataset) to the MFI. iScores were normalized within each tissue section for cross-sample comparisons.

To benchmark iScores, the trained model was applied to two independent EVT scRNA-seq datasets, including second-trimester smooth chorion and placental villi (GSE198373)^40^, term placental tissues from placenta accreta spectrum (PAS) patients and healthy controls (GSE212505)^41^. Gene expression matrices were log-normalized, *z*-score standardized, and used as input to the trained model. Predicted iScores were compared across tissue contexts and disease states to evaluate differences in the predicted EVT invasiveness. To assess the effects of DSC subtypes on neighbouring EVTs, iScores of EVTs in direct spatial proximity to annotated DSC subtypes (within five neighbouring tiles on grids, by Squidpy^75^) were analysed and compared with depth-matched EVTs that were not in spatial proximity to any DSCs. Statistical comparisons were performed using the two-tailed Wilcoxon rank-sum test with Benjamini–Hochberg correction for multiple testing.

### Immunofluorescence

Immunofluorescence was performed as previously described^40,76^. Placenta tissues were fixed in 3% paraformaldehyde in PBS for 30 min, followed by cryoprotection in a sucrose gradient (5%, 10%, 15% in PBS). Tissues were embedded in OCT (Tissue-Tek, Sakura Finetec), sectioned at 10 µm using a Leica cryostat, and mounted on poly-l-lysine-coated slides (Electron Microscopy Sciences). The tissue sections on coverslips were permeabilized using 0.3% Triton X-100 for 10 min and blocked in 1% BSA in PBS for 30 min. Sections were incubated with primary antibodies (Supplementary Table 6) in blocking buffer for 2 h at room temperature, washed four times with PBS, and incubated with Alexa Fluor 488- or 647-conjugated secondary antibodies (1:200; Jackson ImmunoResearch) for 1 h. After PBS washes, nuclei were counterstained with DAPI. Slides were mounted using ProLong Gold Antifade Mountant and stored at 4 °C in the dark prior to imaging. Images were acquired using a Leica DM5000 B microscope under optimized settings.

### Single-molecule fluorescent in situ hybridization with RNAscope

Freshly dissected basal plate samples were fixed in 4% paraformaldehyde for 30 min at room temperature, embedded in OCT, and cryosectioned at 15 μm. Sections were permeabilized and subjected to target retrieval by protease prior to probe hybridization. In situ hybridization was performed using the RNAscope Multiplex Fluorescent Reagent Kit v.2 (Advanced Cell Diagnostics) following the manufacturer’s instructions. Sections were hybridized with a *GPC5*-pecific probe for 2 h at 40 °C in a humidified chamber, followed by signal amplification and detection using TSA-conjugated fluorophores. After hybridization, sections were washed in PBS and processed for immunofluorescence staining with a FITC-conjugated anti-CD94 antibody (BD Biosciences, 555888). Nuclei were counterstained with DAPI. Sections were mounted with ProLong Gold Antifade Mountant (Invitrogen) and stored at 4 °C prior to imaging. Images were acquired using a STELLARIS 5 Confocal Microscope (Leica Microsystems) with consistent acquisition settings across samples. Multiple representative fields were captured per section from three biological replicates. Quantification of *GPC5*^+^ cells was performed in QuPath using automated cell segmentation within defined regions of interest (syncytial aggregates or membrane), with manual verification. Results were reported as the percentage of *GPC5*^+^ cells among total DAPI^+^ nuclei. Statistical significance was assessed using two-tailed Student’s *t*-test.

### In vitro decidualization and mAEA treatment

The HuF cells were collected from human term placenta the decidualized in vitro as described^48^. In brief, the decidual tissues were micro-dissected from the chorionic layer and enzymatically dispersed to reach over 95% purity. The cells are cultured in RPMI medium supplemented with 2% fetal bovine serum and antibiotics. The cells were decidualized for a continuous 7 days by adding medroxyprogesterone acetate (1 μM), oestradiol (10 nM) and prostaglandin E_2_ (1 μM) after reaching 90% confluence. On day 5, 500 nM mAEA or ethanol was added to the culture for 72 h. The conditioned media were collected 24 h after the medium replacement. In parallel experiments, the cells were collected by trypsin digestion on day 7 for 10× Chromium 3′ single-cell RNA sequencing. To identify enriched pathways in decidualized stromal cells following mAEA treatment, gene set enrichment analysis was performed. Genes were ranked by their log-fold changes from differential expression analysis, and pathways were considered significantly enriched at a threshold of FDR < 0.25, as recommended by gene set enrichment analysis guidelines.

### Transwell invasion assay

Transwell invasion assays were performed using 24-well inserts with 8 µm pore-size polycarbonate membranes (Corning Costar, 3422). Membranes were coated with 10 µl of Matrigel (Corning) diluted 1:1 in serum-free DMEM/H-21 supplemented with 2% Nutridoma, 1% Penicillin-Streptomycin, 1% HEPES and 0.1% gentamicin. Trophoblasts isolated from second-trimester basal plate were resuspended in serum-free medium and seeded into the upper chamber at 2.5 × 10^5^ cells per insert. The lower chamber contained conditioned medium from in vitro decidualized HuFs treated with vehicle, mAEA (methanandamide), rimonabant (SRI141716) or mAEA plus rimonabant. Cells were incubated at 37 °C with 5% CO_2_ for 48 h to allow invasion through the Matrigel-coated membrane. Following incubation, inserts were fixed and permeabilized in ice-cold methanol for 20 min, washed with PBS, and stained with DAPI for 5 min at room temperature. Membranes were mounted on slides and imaged at 40× magnification under a fluorescence microscope (Leica Microsystems), with at least 5 non-overlapping fields acquired per insert. Each condition was tested on primarily isolated cytotrophoblasts from three independent placentas. Invaded cells stained with DAPI were counted manually and normalized to the total number of DAPI-positive cells per field. Comparisons between conditions were performed using a two-tailed Student’s *t*-test, with *P* values < 0.05 considered statistically significant.

### Single-cell risk map for pregnancy complications

Single-cell trait relevance scores (TRS) were computed using SCAVENGE by integrating snATAC–seq with GWAS summary statistics of pregnancy complications^50^. Cell-type-specific ATAC peaks were identified using MACS2 (v.2.2.7.1; default)^63^, and cell-by-peak matrices were generated as input for recommended SCAVENGE workflow. Analyses were performed using GWAS datasets for pre-eclampsia (maternal and fetal genomes; EGAD00010001984 and EGAD00010001986)^51^, spontaneous preterm birth (maternal genomes)^52,53^, and sporadic miscarriage (maternal genomes)^54^. Risk-associated cells were defined as cells receiving top 5% TRS scores by permutation testing within SCAVENGE^50^. Genetically vulnerable cell types were determined by the two-tailed Fisher’s exact after Benjamini–Hochberg correction, in which cell types with fewer than 200 cells were excluded. As negative controls, identical analyses using pre-eclampsia GWAS data were performed on adult and prenatal brain single-cell datasets^56,57^ (adult: GSE204684; fetal: https://assets.nemoarchive.org/dat-oiif74w), in which the percentage of genetically vulnerable cells in each cell type was normalized for visualization.

### Ethics statement

All human pregnancy tissue samples were obtained with informed consent and processed in accordance with the Declaration of Helsinki. Study protocols were approved by the Stanford institutional review board (31552, 34745, 48255 and 46584) and University of California, San Francisco institutional review board (11-05530 and 10-00350). All samples were de-identified before processing.

### Reporting summary

Further information on research design is available in the Nature Portfolio Reporting Summary linked to this article.

## Online content

Any methods, additional references, Nature Portfolio reporting summaries, source data, extended data, supplementary information, acknowledgements, peer review information; details of author contributions and competing interests; and statements of data and code availability are available at 10.1038/s41586-026-10316-x.

## Supplementary information

Supplementary Information Supplementary Methods describe the additional details of sample processing and computational analysis not listed in Methods; Supplementary Notes contain the additional information of CODEX imaging and single-cell GWAS analysis not described in the main text.

Reporting Summary

Supplementary Tables Supplementary Tables 1–13 and Supplementary Tables guide

## Source data

Source Data Fig. 1

Source Data Fig. 2

Source Data Fig. 4

Source Data Fig. 5

Source Data Fig. 6

Source Data Extended Data Fig. 1

Source Data Extended Data Fig. 2

Source Data Extended Data Fig. 3

Source Data Extended Data Fig. 4

Source Data Extended Data Fig. 7

Source Data Extended Data Fig. 8

Source Data Extended Data Fig. 9

Source Data Extended Data Fig. 11

Source Data Extended Data Fig. 12

## Data Availability

The COSMOS (Cellular Omics and Spatial Mapping Of States) explorer for data visualization is available at https://cell.ucsf.edu/. Data used in this study can be accessed interactively and downloaded on https://cell.ucsf.edu/snPlacenta/. The raw FASTQ data can be accessed from NIH database of Genotypes and Phenotypes (dbGaP) under controlled access under the accession number phs004305.v1. The data can be accessed by submitting a data access request to the dbGaP Data Access Committee. GWAS data for maternal or fetal compartments in pre-eclampsia can be downloaded from https://ega-archive.org/studies/EGAS00001001266. The full GWAS summary statistics from the meta-analysis of spontaneous preterm birth are available at the Early Growth Genetics Consortium website (https://egg-consortium.org/). Summary statistics of the spontaneous preterm birth cohort from 23andMe can be obtained under an agreement that protects the privacy of the 23andMe participants. Please visit https://research.23andme.com/collaborate/#dataset-access/ for more information and to apply to access the data. The GWAS summary for sporadic miscarriage can be downloaded from http://www.geenivaramu.ee/tools/misc_sumstats.zip. The single-cell datasets used in this study can be accessed from the Gene Expression Omnibus under accessions GSE198373, GSE212505 and GSE204684, and the NeMO archive (https://assets.nemoarchive.org/dat-oiif74w). The source data for visualization can be found in Supplementary Information. Source data are provided with this paper.

## Code availability

The code used for data analysis in this study is available at GitHub (https://github.com/complexdisease/mf.interface).
