# Supplementary material for: Single-cell spatiotemporal dissection of the human maternal–fetal interface
Source: Nature. 2026 Apr 8;653(8113):167–79. doi: 10.1038/s41586-026-10316-x (PMC13149032; doi:10.1038/s41586-026-10316-x)
Supplement: Supplementary file 1 — Supplementary Methods describe the additional details of sample processing and computational analysis not listed in Methods; Supplementary Notes contain the additional information of CODEX imaging and single-cell GWAS analysis not described in the main text. [file 41586_2026_10316_MOESM1_ESM.pdf]

nature portfolio

https://doi.org/10.1038/s41586-026-10316-x

Supplementary information

Single-cell spatiotemporal dissection of the human maternal–fetal interface

In the format provided by the authors and unedited

1. Supplementary Methods.

Tissue acquisition and processing for replicated scRNA-seq

To replicate the identification of DSC3/4 subtypes from independent samples, scRNA-seq was performed on decidual swab samples collected during cesarean deliveries. All study protocols were approved by the University of California, San Francisco Institutional Review Board (IRB #10-00350). Written informed consent was obtained from all participants prior to enrollment. Cesarean sections were performed according to routine clinical protocols. Immediately after placental delivery, the inner uterine surface was sampled using sterile gauze swabs. The first two swabs targeted the anterior uterine wall near the fundus, contralateral to the placental implantation site, while the third swab was taken from the anterior uterus on the same side as the placenta. Each gauze swab was placed into a separate tube containing Iscove's Modified Dulbecco's Medium (IMDM) for immediate preservation and kept on ice until further processing.

Tissue collected on the swabs was minced thoroughly and cryopreserved in 90% DMSO and 10% fetal bovine serum (FBS) in liquid nitrogen. For single-cell dissociation, samples were rapidly thawed in a 37 °C water bath for 2 minutes with gentle agitation, followed by two washes in RPMI-1640 medium supplemented with 10% FBS (RPMI10). Tissues were enzymatically digested in a solution containing 0.28 Wünsch units/mL Liberase 3 (Roche, Cat# 5401119001), 30 µg/mL DNase I (Roche, Cat# 4536282001), and 0.05% trypsin in HBSS with Ca^{2+}/Mg^{2+} for 60 minutes at 37 °C. Gentle mechanical trituration was performed every 10 minutes during the digestion. To reduce cell aggregation, 5 mM EDTA was added during the final 15 minutes of incubation. The resulting cell suspensions were filtered through 100 µm cell strainers, centrifuged, and washed in PBS containing 0.04% BSA. Dead cells were depleted using the Dead Cell Removal Kit (Miltenyi Biotec) according to the manufacturer's instructions. Cell viability and counts were assessed using the Cellaca MX imaging cytometer (Nexcelom Bioscience). Live-cell enriched single-cell suspensions were then processed using the Chromium Single Cell 5′ v1.1 Reagent Kit (10x Genomics) following the manufacturer's protocol. Libraries were sequenced on an Illumina NovaSeq 6000 platform with a target depth of 25,000 reads per cell for gene expression profiling.

Spatial transcriptomic experiments and analysis

1) Library preparation

Tissue fixation and subsequent steps for spatial barcoding and library generation followed the manufacturer's protocols(https://en.stomics.tech/products/stereo-seq-transcriptomics-mIF-solution/list.html). Mounted tissue sections were incubated at 37 °C for 5 minutes, then fixed in pre-chilled methanol (Cat# 34860, Sigma-Aldrich) at −20 °C for 30 minutes. After drying out methanol in a fume hood, tissue sections on the chip were incubated for 20 minutes in the Blocking Solution (with 0.05U/ml RNAse inhibitor added) provided by the manufacturer. The tissue sections were then incubated sequentially with primary antibodies and secondary antibodies diluted in the Blocking Solution for 45 and 30 minutes. After each incubation, slides were washed three times by 0.1X SSC containing 0.05 U/mL RNase inhibitor. After staining with DAPI for 2 minutes, the whole-section images were captured using Zeiss Axio Scan Z1 microscope (at FITC/TRITC wavelength). Tissue sections were then permeated by incubating in permeabilization buffer (Cat# 111KT114, Complete Genomics) at 37°C for 8 minutes. The release RNAs in this step were then reverse transcribed in situ for 2 hours at 45°C. After reverse transcription, tissue sections were washed with 0.1x SSC buffer and digested with tissue removal buffer at 55°C for 10 min. The collected cDNA was denatured and amplified by PCR for 13 cycles. The PCR products were then purified using 0.6 x SPRIselect Bead (Beckman Coulter) and were quantified by Qubit dsDNA High Sensitivity assay kit (cat# Q32854, Invitrogen,). From each sample, 100 ng of purified cDNA was used to construct sequencing libraries with the Stereo-seq library preparation kit (Cat# 111KL114) following the manufacturer's instructions, followed by DNB (DNA Nano Ball) generation and array loading. The resulting DNBs were sequenced on the

DNBSEQ™ T7 platform (Complete Genomics) using a paired-end configuration (50 bp read 1 and 100 bp read 2).

2) Benchmark of data integration and batch correction

To enable joint analysis across individuals and spatial resolutions, single-cell spatial transcriptomic data from all 16 basal plate samples (from total 10 STOmics T chips) were integrated at CellBin resolution. Molecular identifier (MID) counts were log-normalized, and gene dispersion was calculated across the combined dataset. The top 3,000 highly variable genes were selected for dimensional reduction by PCA. To mitigate batch effects arising from differences in sequencing batches, staining protocols, and tissue handling/slides, batch effects were removed using the Harmony workflow, which aligns shared biological structure while preserving biologically meaningful variation[1]. The effectiveness of batch correction was quantitatively assessed using silhouette scores computed before and after Harmony integration (Extended Data Fig.4). Silhouette scores were calculated with respect to batch labels (including tissue slide, sequencing batch, and staining strategy) as well as biologically annotated cell types. A reduction in silhouette scores associated with batch labels after correction was interpreted as evidence of successful batch effect removal[1,2]. Silhouette scores were summarized as mean ± standard deviation across all cells.

3) Cell community detection and co-occurrence analysis

Cell community analysis was performed using the Cell Community Detection (CCD) algorithm implemented in Stereopy (v1.6.1) with default parameters[3]. All tissue sections were jointly analyzed using overlapping sliding windows of 300 pixels. Within each window, the relative abundance of annotated cell types was calculated and represented as a feature vector. Feature vectors from all windows were clustered using the Leiden algorithm after exclusion of low-content windows to identify distinct tissue communities. Each cell was assigned to a community by majority voting across all overlapping windows in which it was included. Identified communities were annotated into six structural categories based on their spatial distribution and cell-type composition.

Cell–cell co-occurrence and neighborhood relationships were quantified using spatial proximity analysis implemented in Stereopy[3]. Pairwise Euclidean distances between all cells were computed from their two-dimensional spatial coordinates, and only cell pairs within 300 pixels (150 μm) were considered. For a given cell type A, co-occurrence with cell type B was defined as the number of A–B neighboring pairs normalized by the total number of A cells, yielding a directional and asymmetric measure. To obtain a symmetric co-occurrence metric, directional scores for A–B and B–A were averaged. Only normalized co-occurrence scores between distinct cell types were visualized and interpreted.

4) Spatial distance quantification to anatomical landmarks

To quantify spatial relationships between individual segmented cells and key anatomical landmarks, we computed distances from each CellBin to the maternal–fetal interface (MFI) and blood vessel walls (BVW) using high-resolution Stereo-seq spatial coordinates (0.5 μm per pixel). The MFI was manually annotated for each tissue section in QuPath (v0.5.1)[4] based on established histological features, supported by immunofluorescence signals and tissue morphology, and independently verified by pathologists. Annotated MFI regions were converted into binary masks and used for distance calculations. For each cell, the shortest Euclidean distance from its centroid to the nearest point along the MFI boundary was computed. Distances were represented as signed values, with positive values indicating the maternal side and negative values indicating the fetal side. Blood vessels were identified based on morphological features in CD31 immunofluorescence or nuclei-stained images and further validated by high expression of endothelial markers (e.g., *PECAM1*, *VWF*, *MMP1*). Vessel boundaries were similarly annotated in QuPath to generate vessel masks. The distance from each cell to the nearest vessel wall was calculated as the shortest Euclidean distance from the centroid to the vessel boundary, with negative values denoting intraluminal localization and positive values indicating increasing distance into the maternal decidua.

Distance calculations were validated by overlaying distance contours onto tissue images. These spatial metrics were subsequently used to analyze gradients of gene expression and cell-type distribution relative to functional tissue landmarks.

Spatial proximity between arterial endothelial cells (aECs) and EVTs was quantified by calculating the Euclidean distance between the centroid of each aEC and its nearest EVT using single-cell–resolved spatial transcriptomic segmentation data. Distances were computed in pixel units and converted to physical distances assuming a spatial resolution of 0.5 μm per pixel. Statistical significance was evaluated using the two-tailed Wilcoxon rank-sum test, with multiple-testing correction applied using the Benjamini–Hochberg procedure.

5) Cell density analysis at blood vessel walls

Stereo-seq data from all 16 tissue sections were analyzed to assess spatial enrichment of a given cell type (e.g. EVTs) near blood vessels. For each identified vessel (total 62 vessels), concentric regions extending up to 200 pixels (100 μm) from the blood vessel walls (BVW) were defined. For comparison, ten control regions with matched area were randomly selected within the maternal decidua from the same tissue section. Cell density was calculated as the number of EVTs per unit area (pixels^2) within BVW-proximal regions and matched controls. Statistical significance was assessed using two-tailed paired Wilcoxon rank-sum tests with Benjamini–Hochberg correction for multiple comparisons.

6) Identification and prediction of arterial endothelial cell states

Maternal arterial endothelial cells (aECs) were identified in Stereo-seq data based on their spatial localization along vessel walls of spiral arteries, expression of canonical endothelial markers and enrichment of adjacent EVTs. High-quality aECs were unsupervised clustered into four transcriptionally distinct states using the Louvain algorithm. To identify minimal gene signatures that distinguish these aEC states, we randomly split cells into a training set (70%) and a held-out test set (30%): we trained a multinomial logistic regression model (scikit-learn v1.4.2). Gene expression values were normalized and z-score scaled prior to model fitting. Feature selection prioritized genes with high discriminative coefficients and robust expression across aECs, resulting in the identification of *PDE3A* and *VIM* as optimal predictors. Classification performance was evaluated on the test set using mean area under the receiver operating characteristic curve (AUROC) for each class. Model robustness was assessed by bootstrapping with 10-fold random splits on test set, and mean AUROC across iterations were reported.

7) Pseudovascularization analysis

To quantify the degree of pseudovascularization in EVTs, an endothelial-like score was computed by averaging the expression of the top 100 differentially expressed genes in maternal vascular endothelial cells from snRNA-seq data. Across samples, this score was calculated using Scanpy with default parameters and standardized within each tissue section. Analyses were restricted to EVTs located within 1,000 pixels (500 μm) of annotated blood vessel walls. EVTs were grouped into distance bins relative to the nearest vessel, and Spearman's correlation was used to assess the relationship between endothelial-like scores and EVT-vessel proximity. For visualization, expression of selected endothelium-associated genes was z-score normalized across distance bins.

CODEX multiplexed immunofluorescence imaging

A Codex microfluidic instrument (Akoya Biosciences Inc., CA, USA) integrated with an inverted fluorescence microscope through a custom stage insert was used to automate Codex buffer exchange and image acquisition. The placenta tissue sections were placed into the stage insert and imaged with 7 cycles of Codex protocol. The first and the last cycles were basically blank cycles and used for image registration and image

alignment. A Keyence BZ-X810 fluorescent microscope configured with 4 fluorescent channels (DAPI, FITC, Cy3, Cy5) and equipped with Nikon objective lens (20x) – CFI Plan Apo 20x/0.75NA was used to capture the images. The raw images were further processed by Codex Analysis Manager (CAM, Akoya Biosciences Inc.). The antibodies panel included barcoded antibodies validated by Akoya Biosciences, where four of them were non-conjugated and purchased from different vendors. Unique oligo barcodes were purchased from Akoya Biosciences for custom conjugation following the manufacturer's protocol https://d1fgrgbaaj3jvk.cloudfront.net/wpcontent/uploads/2020/04/CODEX-User-Manual-Rev-B.0.pdf).

2. Supplementary Notes

CODEX Imaging Provides Validation at the Protein Level

We validated endothelial states transition at the protein level by CODEX (PhenoCycler, Akoya Biosciences) imaging, which utilizes customizable panels of up to 50 antibodies conjugated to oligonucleotide barcode sequences in a single tissue staining reaction to achieve subcellular resolution. Guided by our single-nucleus data, we selected validated CODEX antibodies from the Human Tumor Atlas Network project[5], and confirmed the cell type or cell state specificity of 9 antibodies at the maternal-fetal interface (Extended Data Fig. 6g): Ki-67 (proliferation); CD3 (T cells); CDH1 (epithelium/VCT); PDPN (fibroblasts); VIM (stromal/endothelial cells); CD31 (pan-endothelium); CK18 (trophoblast, strongest in EVTs); CD206 (maternal and fetal macrophages); and PDE3A (arterial endothelium, in conjunction with CD31). CODEX was run on three different second-trimester basal-plate samples (GW 15.2, 19.0, 22.1). All yielded concordant patterns. Subsequent analyses focused on the GW 15.2 sample, with the GW 22.1 dataset as a replication. The GW 19.0 sample was reserved for validation of the spiral-artery data.

CODEX imaging confirmed the spatial organization of all the major cell types (**Fig. 2a**). In villous cores, we detected $CD31^+$ endothelial cells, $CD206^+$ Hofbauer macrophages, and $PDPN^+$ mesenchymal cells. VCTs were $CDH1^+$ (Extended Data Fig. 6h-i), VCTs, SCTs and EVTs were $CK18^+$. Consistent with their elevated CK18 mRNA levels, the latter cells had the brightest signals (Extended Data Fig. 6f-g). Uterine glandular epithelium was also $CK18^+$/$CDH1^+$, but CK18-bright EVTs were readily distinguished by their location: near anchoring villi, in the interstitial zones of the uterine wall, and clustered around maternal vessels. These data were replicated in a parallel analysis of the GW 22.1 sample (Extended Data Fig.7a).

In the GW15.2 sample, CODEX imaging identified two spiral arteries (SA-A and SA-B; **Fig. 3a-e**) with distinct endothelial profiles. SA-A was uniformly lined by $PDE3A^+VIM^-$ endothelial cells (state R0, **Fig. 2m, Fig. 3b-c**). In contrast, SA-B had a heterogeneous pattern: the upper vessel wall contained $PDE3A^-VIM^-$ cells (R1), while the lower wall was dominated by $PDE3A^-VIM^+$ cells (R2), and a few residual $PDE3A^+VIM^-$ cells (R0) remained (**Fig. 3d-e**). Consistent with the spatial transcriptomics data, some R2 cells in SA-B were distant from the vessel wall (**Fig. 3d-e**), suggesting ongoing endothelial displacement (**Fig. 2q**). Despite active EVT remodeling, both vessels retained largely continuous endothelial linings (**Fig. 3b-e**).

CODEX imaging of a GW19 decidual sample revealed a more advanced remodeling stage than SA-A and SA-B, with most endothelial cells displaced by EVTs (**Fig. 3f**). Endothelial cells in an R0 state ($PDE3A^+VIM^-$, green arrows) and an R2 state ($PDE3A^-VIM^+$, yellow arrows) were clearly identifiable (**Fig. 3g-i**). Despite the proximity of EVTs, R0 were adherent to the vessel wall, while R2 cells were detached, likely displaced by EVTs engaged in remodeling (**Fig. 3f-i**). These independent observations validated our spatial transcriptomic data. Immunolocalization analysis of independent biological replicates provided additional protein-level confirmation of these intermediate endothelial states (Extended Data Fig.7b).

To characterize the functional attributes of the R0, R1, and R2 endothelial states, we compared each to caECs (**Fig. 2l**) in our spatial transcriptome data. R0 cells ($PDE3A^{high}VIM^{low}$) showed significant downregulation of genes essential for the integrity of endothelial junctions (GO:0070830, FDR = 1.8e-2), including *CLDN5*, *CDH5*,

and *ACVRL1*, as well as reduced immune signaling, particularly in antigen presentation pathways (GO:0002483, FDR = 1.9e-2) (**Fig. 3j**, Extended Data Fig.7c; Supplementary Table S5). These findings defined R0 as a "primed" state—structurally destabilized and immunologically less responsive, yet retaining arterial identity ($CD31^+PDE3A^+$) and attachment to the vessel wall (**Fig. 2q, Fig. 3**). R1 cells (*PDE3A*^{low}*VIM*^{low}) further downregulated *PDE3A* and other arterial markers (*EFNB2*, *HEY1*, *DLL4*), indicating loss of arterial identity (**Fig. 3k**), and showed signs of detachment from the vessel wall (**Fig. 2q**). The R2 state, the terminal phase of EVT-mediated endothelial remodeling, was associated with apoptotic gene activation (**Fig. 2n**, Extended Data Fig.7d) and detachment from the vessel wall (**Fig. 2q, Fig. 3f-i**). Because cell adhesion and junction genes are down-regulated (R0) and signs of vessel wall detachment appear (R1) prior to the induction of apoptosis (R2), the R2 state is most consistent with the process of anoikis. To assess whether endothelial state transitions were autonomously regulated or influenced by proximity to EVTs, we aggregated all samples and compared the Euclidean distance from each endothelial cell to the nearest EVT(s). As shown in Extended Data Fig.7e, caECs were located furthest from EVTs, while R1 and R2 states exhibited progressively shorter distances, suggesting a spatial gradient in which endothelial cells transition toward EVT proximity. This pattern supports our model in which increasing contact with EVTs drives sequential endothelial changes: from arterial identity loss (R1), then to apoptosis (R2). A summary of the model is presented in **Fig. 3l**.

Under this model, SA-A in **Fig. 3b-c** was dominated by R0 cells, reflecting a primed but largely intact endothelial lining. In contrast, SA-B showed a more advanced remodeling stage, with R1 cells along the upper wall and R2 cells along the lower wall undergoing displacement by EVTs. The artery shown in **Fig. 3f** represents a near-terminal stage, where EVTs have extensively replaced the endothelial lining. Collectively, our analysis defines a progressive, stepwise sequence of endothelial transitions modulated by EVT proximities, suggesting that disruption of this sequence may compromise the remodeling process.

Integrating GWAS and Single-Nuclei Data Identified Affected Cell Types in Major Pregnancy Complications

To demonstrate the translational value of our single cell compilation, we leveraged the data to guide our analysis of large-scale patient genomes with major pregnancy complications: preeclampsia, spontaneous preterm births, and sporadic miscarriages. These conditions likely have a significant genetic basis, but genome-wide association studies (GWAS) have implicated very few genomic loci. Thus, their molecular etiologies remain largely elusive.

More than 90% of disease-associated variants fall in noncoding regions, resulting in epigenetic alterations in active regulatory elements and, consequently, aberrant gene expression[6,7]. Thus, it is possible to determine whether a cell type-specific open chromatin is enriched for disease-associated genomic variants. The recently developed SCAVENGE framework integrates GWAS with snATAC-seq data to discover disease-associated cell types[8]. The algorithm identifies single-nucleus open chromatin regions that are enriched for common variants with increased GWAS risk, revealing the cell types most vulnerable to a given disease. We recently leveraged this framework and successfully identified vulnerable cell types to major neurodevelopmental disorders[9].

First, we applied this approach to preeclampsia, a common and potentially life-threatening pregnancy complication that manifests as a sudden rise in maternal blood pressure and the appearance of protein in the

urine. Both signs are the result of maternal vascular damage. This syndrome has a genetic component with heritability as high as 60%; 35%, and 20% are potentially contributed by the maternal and fetal genomes, respectively[10]. We analyzed a large GWAS preeclampsia study[11], and only considered subjects with European ancestry. This study utilized 10.8 million genomic variants genotyped in 10,255 maternal cases and 10,255 matched female controls, together with 10.4 million variants in 7,259 affected fetal genomes (offspring of preeclamptic pregnancies) and 7,259 matched fetal controls. A standard GWAS analysis yielded only two significant SNPs from the maternal genomes and one from the fetal genomes. Their moderate effect sizes (OR<1.3) cannot explain pre-eclampsia heritability observed in population studies.

To identify cellular origins of preeclampsia population genetic risk, we performed SCAVENGE to analyze this preeclampsia GWAS dataset with our snATAC-seq map. We paired maternal genomes with maternal cells, and fetal genomes with fetal cells. SCAVENGE computed the trait relevance score (TRS) for each cell (**Fig. 6a**) and identified 6,221 maternal and 8,232 fetal cells as significantly associated with preeclampsia by permutation analysis. The cells (maternal and fetal) accounted for 7.5% of all those identified (14,453/191,735). Next, we performed enrichment analyses by Fisher's exact test separately in the maternal and fetal compartments, identifying each cell (sub)type for overrepresentation of preeclampsia-associated cells. Among all fetal cell types, only EVTs (all subtypes) displayed a significant enrichment for preeclampsia-associated cells (FDR≤1e-4, **Fig. 6b**) with iEVTs manifesting the strongest enrichment. In contrast, multiple maternal cell types were significantly associated with the genetic risk of preeclampsia. Among the DSC subtypes (**Fig. 5a**), only DSC3 cells, the terminal state of differentiation Path A (**Fig. 5b**), displayed significant enrichment in preeclampsia (**Fig. 6c**). A subset of vascular components also had significant risk enrichments: arterial endothelium (but not venous endothelium), perivascular smooth muscle and perivascular fibroblasts (**Fig. 6c**). Moreover, the enrichment of T cells (**Fig. 6c**) was evidence of maternal immune involvement. Despite the proposed role of decidual macrophage and NK cells in preeclampsia[12,13], population genetic risk was not enriched in these cell types. The association of a unique subset of endometrial epithelial cells was of particular interest (**Fig. 6c**). Many of these cells significantly expressed stem cell markers (OCT4 [*POU5F1*] and *LGR5*, Extended Data Fig. 12a-d)**,** and their presence was limited to early gestation samples. This finding associated early gestation events with genetic etiologies of preeclampsia and suggested an endometrial contribution to the origin of preeclampsia[14]. A decidualization defect has been implicated as well[15].

For independent validation, we examined 151 genes associated with preeclampsia curated by DisGeNet database[16], which contains entries from numerous sources, GWAS as well as transcriptome and biomarker studies. Querying their expression using our reference single-nucleus data showed specific enrichments specifically in the cell types identified in our analysis (Extended Data Fig. 12e). Therefore, this independent comparison confirmed the effectiveness of our approach and highlighted the vulnerable cell types associated with the population genetics of preeclampsia.

As negative controls, we paired the fetal and maternal GWAS datasets with fetal and adult single-cell brain transcriptomes[9,17], respectively, and repeated the analysis. No fetal or adult brain cells showed significant enrichment for preeclampsia risk (Extended Data Fig. 12f-g**)**, supporting the specificity and robustness of our study.

The SCAVENGE analysis was performed for spontaneous preterm labor and spontaneous miscarriage. Because only maternal genomes were available for both conditions, we limited our analysis to maternal cell types. For spontaneous preterm labor, we analyzed GWAS data from 3,331 cases (GW<37) and 37,803 matched female controls[18]. For sporadic miscarriage, we analyzed GWAS data from 49,996 cases and 174,109 matched female controls[19]. In both cases, only the *POU5F1*$^+$*LGR5*$^+$ endometrial epithelial cells displayed significant enrichment for genetic risk at a population level (**Fig. 6d-e**). We replicated the preterm birth analysis in an independent cohort of 233,290 women, including 15,419 cases[20], and again identified endometrial epithelial cells as the genetically vulnerable population (Extended Data Fig. 12h). The shared cellular basis likely explains the clinical associations between spontaneous preterm labor and miscarriage[21,22]. Given that this cell type is also implicated in preeclampsia, our results support the concept of "endometrium spectrum disorders"[23], which suggests that many pregnancy complications are associated with a continuum of dysregulated endometrial functions. In this regard, we theorize that *POU5F1*$^+$*LGR5*$^+$ epithelial cells play a particularly important deterministic role.

References

1 Korsunsky, I. *et al.* Fast, sensitive and accurate integration of single-cell data with Harmony. *Nat Methods* **16**, 1289-1296, doi:10.1038/s41592-019-0619-0 (2019).

2 Luecken, M. D. *et al.* Benchmarking atlas-level data integration in single-cell genomics. *Nat Methods* **19**, 41-50, doi:10.1038/s41592-021-01336-8 (2022).

3 Fang, S. *et al.* Stereopy: modeling comparative and spatiotemporal cellular heterogeneity via multi-sample spatial transcriptomics. *Nat Commun* **16**, 3741, doi:10.1038/s41467-025-58079-9 (2025).

4 Bankhead, P. *et al.* QuPath: Open source software for digital pathology image analysis. *Sci Rep* **7**, 16878, doi:10.1038/s41598-017-17204-5 (2017).

5 Rozenblatt-Rosen, O. *et al.* The Human Tumor Atlas Network: Charting Tumor Transitions across Space and Time at Single-Cell Resolution. *Cell* **181**, 236-249, doi:10.1016/j.cell.2020.03.053 (2020).

6 Corradin, O. & Scacheri, P. C. Enhancer variants: evaluating functions in common disease. *Genome Med* **6**, 85, doi:10.1186/s13073-014-0085-3 (2014).

7 Schaub, M. A., Boyle, A. P., Kundaje, A., Batzoglou, S. & Snyder, M. Linking disease associations with regulatory information in the human genome. *Genome Res* **22**, 1748-1759, doi:10.1101/gr.136127.111 (2012).

8 Yu, F. *et al.* Variant to function mapping at single-cell resolution through network propagation. *Nat Biotechnol* **40**, 1644-1653, doi:10.1038/s41587-022-01341-y (2022).

9 Wang, L. *et al.* Molecular and cellular dynamics of the developing human neocortex. *Nature*, doi:10.1038/s41586-024-08351-7 (2025).

10 Rana, S., Lemoine, E., Granger, J. P. & Karumanchi, S. A. Preeclampsia: Pathophysiology, Challenges, and Perspectives. *Circ Res* **124**, 1094-1112, doi:10.1161/CIRCRESAHA.118.313276 (2019).

11 Steinthorsdottir, V. *et al.* Genetic predisposition to hypertension is associated with preeclampsia in European and Central Asian women. *Nat Commun* **11**, 5976, doi:10.1038/s41467-020-19733-6 (2020).

12 Hiby, S. E. *et al.* Combinations of maternal KIR and fetal HLA-C genes influence the risk of preeclampsia and reproductive success. *J Exp Med* **200**, 957-965, doi:10.1084/jem.20041214 (2004).

13 Faas, M. M., Spaans, F. & De Vos, P. Monocytes and macrophages in pregnancy and pre-eclampsia. *Front Immunol* **5**, 298, doi:10.3389/fimmu.2014.00298 (2014).

14 Conrad, K. P. Evidence for Corpus Luteal and Endometrial Origins of Adverse Pregnancy Outcomes in Women Conceiving with or Without Assisted Reproduction. *Obstet Gynecol Clin North Am* **47**, 163-181, doi:10.1016/j.ogc.2019.10.011 (2020).

15 Munoz-Blat, I. *et al.* Multi-omics-based mapping of decidualization resistance in patients with a history of severe preeclampsia. *Nat Med* **31**, 502-513, doi:10.1038/s41591-024-03407-7 (2025).

16 Pinero, J. *et al.* DisGeNET: a comprehensive platform integrating information on human disease-associated genes and variants. *Nucleic Acids Res* **45**, D833-D839, doi:10.1093/nar/gkw943 (2017).

17 Zhu, K. *et al.* Multi-omic profiling of the developing human cerebral cortex at the single-cell level. *Sci Adv* **9**, eadg3754, doi:10.1126/sciadv.adg3754 (2023).

18 Zhang, G. *et al.* Genetic Associations with Gestational Duration and Spontaneous Preterm Birth. *N Engl J Med* **377**, 1156-1167, doi:10.1056/NEJMoa1612665 (2017).

19 Laisk, T. *et al.* The genetic architecture of sporadic and multiple consecutive miscarriage. *Nat Commun* **11**, 5980, doi:10.1038/s41467-020-19742-5 (2020).

20 Sole-Navais, P. *et al.* Genetic effects on the timing of parturition and links to fetal birth weight. *Nat Genet* **55**, 559-567, doi:10.1038/s41588-023-01343-9 (2023).

21 Swingle, H. M., Colaizy, T. T., Zimmerman, M. B. & Morriss, F. H., Jr. Abortion and the risk of subsequent preterm birth: a systematic review with meta-analyses. *J Reprod Med* **54**, 95-108 (2009).

22 Oliver-Williams, C., Fleming, M., Wood, A. M. & Smith, G. Previous miscarriage and the subsequent risk of preterm birth in Scotland, 1980-2008: a historical cohort study. *BJOG* **122**, 1525-1534, doi:10.1111/1471-0528.13276 (2015).

23 Conrad, K. P., Rabaglino, M. B. & Post Uiterweer, E. D. Emerging role for dysregulated decidualization in the genesis of preeclampsia. *Placenta* **60**, 119-129, doi:10.1016/j.placenta.2017.06.005 (2017).
