# Supplementary material for: Single-cell spatiotemporal dissection of the human maternal–fetal interface
Source: Nature. 2026 Apr 8;653(8113):167–79. doi: 10.1038/s41586-026-10316-x (PMC13149032; doi:10.1038/s41586-026-10316-x)
Supplement: Supplementary file 2 — Reporting Summary [file 41586_2026_10316_MOESM2_ESM.pdf]

nature portfolio

Corresponding author(s): Jingjing Li

Last updated by author(s): Dec 20, 2025

Reporting Summary

Nature Portfolio wishes to improve the reproducibility of the work that we publish. This form provides structure for consistency and transparency in reporting. For further information on Nature Portfolio policies, see our Editorial Policies and the Editorial Policy Checklist.

Statistics

For all statistical analyses, confirm that the following items are present in the figure legend, table legend, main text, or Methods section.

n/a	Confirmed	
☐	☒	The exact sample size (*n*) for each experimental group/condition, given as a discrete number and unit of measurement
☐	☒	A statement on whether measurements were taken from distinct samples or whether the same sample was measured repeatedly
☐	☒	The statistical test(s) used AND whether they are one- or two-sided<br>*Only common tests should be described solely by name; describe more complex techniques in the Methods section.*
☐	☒	A description of all covariates tested
☐	☒	A description of any assumptions or corrections, such as tests of normality and adjustment for multiple comparisons
☐	☒	A full description of the statistical parameters including central tendency (e.g. means) or other basic estimates (e.g. regression coefficient) AND variation (e.g. standard deviation) or associated estimates of uncertainty (e.g. confidence intervals)
☐	☒	For null hypothesis testing, the test statistic (e.g. *F*, *t*, *r*) with confidence intervals, effect sizes, degrees of freedom and *P* value noted<br>*Give P values as exact values whenever suitable.*
☒	☐	For Bayesian analysis, information on the choice of priors and Markov chain Monte Carlo settings
☒	☐	For hierarchical and complex designs, identification of the appropriate level for tests and full reporting of outcomes
☐	☒	Estimates of effect sizes (e.g. Cohen's *d*, Pearson's *r*), indicating how they were calculated

Our web collection on statistics for biologists contains articles on many of the points above.

Software and code

Policy information about availability of computer code

Data collection	No custom code was generated for data collection
Data analysis	The Code availability can be found on https://github.com/complexdisease/mf.interface; Detailed analysis methods can be found in the Methods section of the manuscript. Packages and software used include: Cell Ranger ARC suite (v.2.0.0), MACS2 (v2.2.7), Signac (vl.10.0), Seurat v4.3.0, chromVAR (vl.16.0), squidpy (vl.2.3), scanpy (v1.10), CellChat (vl.6.1), CellOracle (v0.16.0), gcta, palantir (v1.3.3), souporcell, Cell Ranger suite (v.7.1.0), SCAVENGE (vl.0.2), SAW (v8.1), Stereopy (v1.0.0), harmonypy.

For manuscripts utilizing custom algorithms or software that are central to the research but not yet described in published literature, software must be made available to editors and reviewers. We strongly encourage code deposition in a community repository (e.g. GitHub). See the Nature Portfolio guidelines for submitting code & software for further information.

Data

Policy information about availability of data

All manuscripts must include a data availability statement. This statement should provide the following information, where applicable:

- Accession codes, unique identifiers, or web links for publicly available datasets
- A description of any restrictions on data availability
- For clinical datasets or third party data, please ensure that the statement adheres to our policy

The COSMOS (Cellular Omics and Spatial Mapping Of States) explorer for data visualization is available at https://cell.ucsf.edu/. Data used in this study can be

accessed interactively and downloaded on https://cell.ucsf.edu/snPlacenta/. The raw FASTQ data can be accessed from NIH database of Genotypes and Phenotypes (dbGaP) under controlled access under the accession number phs004305.v1. The data can be accessed by submitting a Data Access Request to the dbGaP Data Access Committee. GWAS data for maternal or fetal compartments in preeclampsia can be downloaded from https://ega-archive.org/studies/EGAS00001001266. The full GWAS summary statistics from the meta-analysis of sPTB are available at the EGG website (https://egg-consortium.org/). Summary statistics of the sPTB cohort from 23andme can be obtained under an agreement that protects the privacy of the 23andMe participants. Please visit https://research.23andme.com/collaborate/#dataset-access/ for more information and to apply to access the data. The GWAS summary for sporadic miscarriage can be downloaded from http://www.geenivaramu.ee/tools/misc_sumstats.zip. The single-cell datasets used in this study can be accessed from GEO under accession GSE198373, GSE212505, and GSE204684, and NeMO archive https://assets.nemoarchive.org/dat-oiif74w. The source data for visualization can be found in Supplementary Information.

Research involving human participants, their data, or biological material

Policy information about studies with human participants or human data. See also policy information about sex, gender (identity/presentation), and sexual orientation and race, ethnicity and racism.

Reporting on sex and gender	Sex and gender were not used in any scenario as criteria for sample collection. The gender of the donor included in this study is based on self-reporting and confirmed by post-hoc analyses. Conclusions are not biased by sample sex.
Reporting on race, ethnicity, or other socially relevant groupings	No race, ethnicity or socially relevant groupings were performed in this study.
Population characteristics	We collected placenta samples from gestational week 5 to term. The detailed information of population characteristics can be found in Supplementary Table 1 and 4. No population characteristics except for gestational stages were used in this study.
Recruitment	De-identified tissue samples were collected with previous patient consent in strict observance of the legal and institutional ethical regulations. This was performed by the clinic and only samples without pregnancy complications were selected in this study.
Ethics oversight	All human pregnancy tissue samples were obtained with informed consent and processed in accordance with the Declaration of Helsinki. Study protocols were approved by the Stanford institutional review board (31552, 34745, 48255 and 46584), and UCSF institutional review board (11-05530 and 10-00350). All data were de-identified before processing and analyzed.

Note that full information on the approval of the study protocol must also be provided in the manuscript.

Field-specific reporting

Please select the one below that is the best fit for your research. If you are not sure, read the appropriate sections before making your selection.

☒ Life sciences ☐ Behavioural & social sciences ☐ Ecological, evolutionary & environmental sciences

For a reference copy of the document with all sections, see nature.com/documents/nr-reporting-summary-flat.pdf

Life sciences study design

All studies must disclose on these points even when the disclosure is negative.

Sample size	Because this is not a case-control study design, traditional sample size calculation is not applicable to this study. We included as many samples as possible in each developmental stage to identify more diverse cell type populations.
Data exclusions	NA. Sample metadata are provided in Supplementary Information. Individuals with pregnancy complications or fetal chromosomal abnormalities are excluded.
Replication	Number of biological replicates were described in the manuscripts
Randomization	The permutation test was performed to calculate the p values. Samples were analyzed and sequenced in random orders.
Blinding	Sample processing was blinded to sample metadata information (gestational age, etc.).

Reporting for specific materials, systems and methods

We require information from authors about some types of materials, experimental systems and methods used in many studies. Here, indicate whether each material, system or method listed is relevant to your study. If you are not sure if a list item applies to your research, read the appropriate section before selecting a response.

Materials & experimental systems

n/a	Involved in the study
☐	☒ Antibodies
☐	☒ Eukaryotic cell lines
☒	☐ Palaeontology and archaeology
☒	☐ Animals and other organisms
☒	☐ Clinical data
☒	☐ Dual use research of concern
☒	☐ Plants

Methods

n/a	Involved in the study
☒	☐ ChIP-seq
☒	☐ Flow cytometry
☒	☐ MRI-based neuroimaging

Antibodies

Antibodies used: The details of the antibodies information can be found in Supplementary table S5, including the catalogue number, clone name and the dilution used

Validation: All antibodies used CODEX and immunostaining analysis have been validated by the manufacturer to be suitable for the application

Eukaryotic cell lines

Policy information about cell lines and Sex and Gender in Research

Cell line source(s): Primary maternal decidua

Authentication: The primary cell line has been authenticated by experiments in previous publication (PMID: 32990600)

Mycoplasma contamination: No mycoplasma has been detected.

Commonly misidentified lines (See ICLAC register): Misidentification are not applicable to the primary cell line.

Plants

Seed stocks: N/A

Novel plant genotypes: N/A

Authentication: N/A
