# Supplementary material for: Single-cell spatiotemporal dissection of the human maternal–fetal interface
Source: Nature. 2026 Apr 8;653(8113):167–79. doi: 10.1038/s41586-026-10316-x (PMC13149032; doi:10.1038/s41586-026-10316-x)
Supplement: Supplementary file 3 — Supplementary Tables 1–13 and Supplementary Tables guide [file 41586_2026_10316_MOESM3_ESM.zip › Supplementary Table Guide.pdf]

Supplementary Table Guide

Supplementary Table 1. a). Summary of published single-cell studies profiling the human placenta/decidua. **b**). A glossary of abbreviation used in this study. **c**). Metadata for samples profiled by single-nucleus multiome sequencing in this study. **d**). Quality control (QC) summary of estimated nucleus counts as determined by CellRanger ARC. **e**) Summary metrics for nuclei that passed QC filters.

Supplementary Table 2. a). Top-ranked marker genes identified for each major cell type. **b**). List of experimentally validated enhancers with differential activity across placental/decidual cell types.
**CAGE enhancers are from FANTOM5 collection (PMID: 28850107)*

Supplementary Table 3. a). Transcription factors specific to trophoblast lineage differentiation. **b**). EVT-specific transcription factor–target gene regulatory interactions. **c**). SCT-specific transcription factor–target gene regulatory interactions.
**All interactions are from CellOracle analysis.*

Supplementary Table 4. a). Metadata of tissue sections profiled using STOmics (Stereo-seq). **b**). Summary statistics of Stereo-seq data prior to quality control. **c**). Post-QC summary of Stereo-seq spatial transcriptomic data. **d**). Spatial density quantification of EVTs in proximity to maternal blood vessel walls**. e).** Endothelial cell state classification performance using *PDE3A* and *VIM* expression by bootstrapping.

Supplementary Table 5. a). List of top-ranked genes downregulated in R0 aECs vs canonical aEC. **b**). List of top-ranked genes downregulated in R1 aECs vs canonical aEC. **c**). List of top-ranked genes downregulated in R2 aECs vs canonical aEC. **d**). Gene ontology enrichment analysis of downregulated (R0) genes in comparison to canonical aECs. **e).** Gene ontology enrichment analysis of downregulated (R2) genes in comparison to canonical aECs.
**Genes with DEG scores greater than 10 were selected for GO analysis except for ribosomal genes.*

Supplementary Table 6. a). The antibody and marker panel used for CODEX multiplexed imaging. **b**). List of antibodies and conjugates used for immunofluorescence staining.

Supplementary Table 7. a). Top-ranked differentially expressed genes in endovascular EVTs (eEVT) versus interstitial EVTs (iEVT). **b**). Top-ranked differentially expressed genes in perivascular EVTs (pEVT) versus interstitial EVTs (iEVT). **c**). GO enrichment of marker genes specific to eEVT. **d**). GO enrichment of marker genes specific to pEVT. **e**). GO enrichment of marker genes specific to iEVT populations.
**Non-ribosomal genes receiving DEG scores over 20 were selected for GO analysis.*

Supplementary Table 8. a). Top endothelial-enriched genes used to score endovascular EVT resemblance to vascular endothelium. **b).** Top-ranked differentially expressed genes between EVTs with high versus low iScores for GO analysis. **c).** GO enrichment of upregulated genes in high iScore EVTs. **d).** GO enrichment of downregulated genes in high iScore EVTs. **e).** List of model-selected genes and their learned coefficients for predicting EVT invasiveness. Positive and negative coefficients indicate pro- and anti-invasive effects on EVT invasiveness, respectively.
**Top 100 mVEC-enriched genes except for ribosomal and mitochondrial genes were selected for scoring; For GO analysis, genes receiving the highest DEG scores were selected except for ribosomal genes*

Supplementary Table 9. **a**). Gene expression dynamics during the VCT-to-SCT transition. **b**). Quantification of *GPC5*+ SCT-B cells in RNAscope experiments. **c**). Quantification of *GPC5*+ SCT-B cells in STOmics spatial transcriptomics.

Supplementary Table 10. a). Top-ranked differential gene expression between Path A and Path B decidual stromal cells (DSCs) for GO analysis. **b).** GO terms enriched among genes upregulated in Path A DSCs. **c).** GO terms enriched among genes upregulated in Path B DSCs. **d).** Top-ranked differentially expressed genes between DSC3.1 and DSC3.2 subtypes.
**All non-ribosomal genes receiving DEG scores over 20 were selected for GO analysis.*

Supplementary Table 11. a). Differentially expressed genes in DSC3 cells following mAEA treatment. **b).** Differentially expressed genes in DSC4 cells following mAEA treatment.

Supplementary Table 12. Quantification of trophoblast invasion in Transwell assays. The cell counts are normalized to the vehicle controls in percentage.

Supplementary Table 13. a). GWAS-based preeclampsia relevance scores across fetal cell types. **b).** GWAS-based preeclampsia relevance scores across maternal cell types. **c).** Relevance scores for spontaneous preterm birth (sPTB) across maternal cell types. **d).** Relevance scores for sporadic miscarriage across maternal cell types. **e).** List of DisGeNET-curated genes in preeclampsia. **f**). GWAS-based relevance scores for preeclampisa across prenatal brain cell types. **g**). GWAS-based relevance scores for preeclampisa across adult brain cell types.
**snATAC-seq datasets were from Wang et al.,2025 (PMID: 39779846, f), and Zhu et al., 2023, PMID37824614, g)*
